# Melatonin ameliorates necrotizing enterocolitis by preventing Th17/Treg imbalance through activation of the AMPK/SIRT1 pathway

**DOI:** 10.7150/thno.45862

**Published:** 2020-06-19

**Authors:** Fei Ma, Hu Hao, Xiaoyan Gao, Yao Cai, Jialiang Zhou, Puping Liang, Junjian Lv, Qiuming He, Congcong Shi, Dandan Hu, Bowei Chen, Lixin Zhu, Xin Xiao, Sitao Li

**Affiliations:** 1 Department of Pediatrics, The Sixth Affiliated Hospital, Sun Yat-sen University, Guangzhou, 510655, China.; 2 Guangdong Institute of Gastroenterology, The Sixth Affiliated Hospital, Sun Yat-sen University, Guangzhou, 510655, China.; 3 Department of Neonatology, the Foshan Women and Children hospital, Foshan, 528000, China.; 4 Department of Neonatal Surgery, Guangdong Women and Children Hospital, Guangzhou, 511400, China.; 5 MOE Key Laboratory of Gene Function and Regulation, School of Life Sciences, Sun Yat-sen University, Guangzhou, 510275, China.; 6 Congenital Diaphragmatic Hernia Study and Collaborative Group of Fetal Care Center, Municipal Women and Children's Medical Center, Guangzhou, 510623, China.; 7 Zhongshan School of medicine, Sun Yat-sen University, Guangzhou, 510080, China.

**Keywords:** melatonin, necrotizing enterocolitis, Th17/Treg imbalance, AMPK/SIRT1 pathway, intestine

## Abstract

Necrotizing enterocolitis (NEC) is a severe gastrointestinal disease affecting premature infants. Mounting evidence supports the therapeutic effect of melatonin on NEC, although the underlying mechanisms remain unclear.

**Methods:** NEC was induced in 10-day-old C57BL/6 pups via hypoxia and gavage feeding of formula containing enteric bacteria, and then, mice received melatonin, melatonin + recombinant IL-17, melatonin + anti-CD25 monoclonal antibody, melatonin + Ex-527, or melatonin + Compound C treatment. Control mice were left with their dams to breastfeed and vehicle-treated NEC pups were used as controls for treatment. Ileal tissues were collected from mice and analyzed by histopathology, immunoblotting, and flow cytometry. FITC-labeled dextran was administered to all surviving pups to evaluate gut barrier function by fluorometry. We used molecular biology and cell culture approaches to study the related mechanisms in CD4^+^ T cells from umbilical cord blood.

**Results:** We demonstrated that melatonin treatment ameliorates disease in an NEC mouse model in a manner dependent on improved intestinal Th17/Treg balance. We also showed that melatonin blocks the differentiation of pathogenic Th17 cells and augments the generation of protective Treg cells in vitro. We further demonstrated that the Th17/Treg balance is influenced by melatonin through activation of AMPK in the intestine, in turn promoting SIRT1 activation and stabilization.

**Conclusions:** These results demonstrate that melatonin-induced activation of AMPK/SIRT1 signaling regulates the balance between Th17 and Treg cells and that therapeutic strategies targeting the Th17/Treg balance via the AMPK/SIRT1 pathway might be beneficial for the treatment of NEC.

## Introduction

Necrotizing enterocolitis (NEC) constitutes a gastrointestinal disease of premature infants with high morbidity and mortality that is characterized by an exaggerated inflammatory response and necrosis in the intestine 1-3. Accumulating evidence suggests that disruption of the T helper 17 (Th17) cell and FoxP3^+^ regulatory T (Treg) cell balance is an important factor underlying the powerful inflammatory response in the intestine 4-7. In NEC, the T cell imbalance is characterized by a proinflammatory Th17 cell profile along with a reduction in Treg cells 5-7. Although we and others have recently demonstrated that imbalances in the Th17/Treg ratio could be mitigated by anti-IL-6R or anti-IL-17RC antibody treatment 5, 6, the mechanisms underlying such imbalances are not yet fully understood.

Sirtuin 1 (SIRT1), which belongs to the family of class III histone deacetylases, plays an important role in various physiological processes 8-10. A recent study revealed that the conditional knockout (KO) of SIRT1 in T cells results in decreased Th17 differentiation and ameliorates experimental autoimmune encephalomyelitis (EAE) 11. Moreover, conditional SIRT1 KO in T cells was further shown to induce Treg differentiation, thereby prolonging allograft survival 12, 13. However, a recent study on patients with acute graft-versus-host disease (aGVHD) revealed contradictory results, as SIRT1 deficiency in CD4^+^ T cells played a crucial role in upregulating STAT3 phosphorylation, in turn increasing Th17 differentiation and inducing aGVHD 14. This latter finding was further supported by a study on SIRT1-deficient mice, in which T cells failed to maintain tolerance leading to the development of severe EAE 15. Thus, although decreased levels of SIRT1 have been observed in the inflamed intestinal mucosa of patients with NEC 16, the relationship between this phenomenon and Th17/Treg imbalance remains unclear.

Like pinealocytes, enterochromaffin cells throughout the gut are highly effective in producing melatonin. Further, the levels of intestinal melatonin peak at birth and then decline to stable levels at the age of 21 d in mice 17. Studies indicate that melatonin can regulate the responses of Th1, Th2, Th17, and Treg cells, which are associated with various autoimmune- and infection-related diseases, in addition to cancers 18. Notably, melatonin treatment significantly alleviates NEC in both humans and rats 19, 20. However, to our knowledge whether the potential therapeutic effects of melatonin on NEC are mediated through intestinal Th17/Treg differentiation has not yet been determined. Given the essential role of imbalanced Th17/Treg cells in the development and progression of NEC, we speculated that melatonin treatment might promote the activation of SIRT1 and subsequently prevent the imbalance of intestinal Th17/Treg cells. Thus, the aim of this study was to test this hypothesis and to elucidate the underlying mechanisms in NEC.

## Materials and Methods

### Mice and experimental design of NEC

C57BL/6 mice were obtained from the Experimental Animal Center of Southern Medical University (Guangzhou, China) and were bred in a specific pathogen-free facility. All animal experiments were approved by the Institutional Animal Care and Use Committee of The Sixth Affiliated Hospital, Sun Yat-sen University (protocol number: 20191028-005). NEC was induced as described in our previous report 5, 21 in 10-day-old mouse pups via gavage feeding (five times daily) of formula [Similac Advance infant formula (Abbott Nutrition, Columbus, OH, USA): Esbilac (PetAg, Hampshire, IL, USA) milk replacer for puppies, 2:1] containing enteric bacteria from a patient with surgical NEC (12.5 µL original stool slurry in 1 mL formula). Mice were simultaneously exposed to hypoxic conditions (5% O_2_, 95% N_2_) for 10 min twice per day in a modular chamber (Billups-Rothenberg, San Diego, CA, USA) for 4 days.

The experimental pups were treated with melatonin once daily 1 h prior to the NEC procedure until the end of the experiment. Each mouse was intraperitoneally (i.p.) injected with melatonin (Cat #S1204, Selleck Chemicals, Shanghai, China) at 10 mg/kg body weight in a total volume of 100 μL; the melatonin was dissolved in a vehicle consisting of < 25% ethanol in phosphate-buffered saline (PBS; Cat #18912014, Thermo Fisher Scientific, PA, USA). For exogenous IL-17 administration experiments, mice were injected with 100 ng of rIL-17A (Cat #210-17, Peprotech, Rocky Hill, NJ, USA) once daily on postnatal day (P) 9 to P12 via i.p. injection. In Treg cell depletion experiments, aCD25 mAb (Cat #102002, BioLegend, San Diego, CA USA, clone: PC61) or control IgG1 (cIgG1, Cat #401902, BioLegend) was administered to mice at 10 µg/mouse via i.p. injection on P2 and P8. rIL-17A, aCD25 mAb, and cIgG1 were dissolved in PBS. To prevent SIRT1 and AMPK activation, Ex-527 (2 mg/kg body weight; Cat #S1541, Selleck Chemicals) and Compound C (20 mg/kg body weight; Cat #S7840, Selleck Chemicals) were also dissolved in the same vehicle as melatonin, and mice were i.p. injected with 100 μL of either agent once daily on P9, P10, P11, and P12. Control mice (BF group) were left with their dams to breastfeed and vehicle-treated NEC pups (VEH group) were i.p. injected with the vehicle as a control for melatonin (MEL group), melatonin + rIL-17 (NEC + MEL+ rIL-17 group), melatonin + aCD25 mAb (MEL+ aCD25 mAb group), melatonin + Ex-527 (MEL+ Ex-527 group), and melatonin + Compound C (MEL+ CC group) treatment. These pups were monitored closely and weighed on P10, P12, and P14. Animals were euthanized on P14 or earlier if they demonstrated NEC signs.

### Intestinal mucosa permeability assay

To measure gut mucosal permeability, we administered fluorescein isothiocyanate (FITC)-labeled dextran (FD70; Cat #60842-46-8, Sigma-Aldrich, St. Louis, MO, USA) as described previously 22. Briefly, all surviving mouse pups at the end of the experiments were gavaged with 750 mg/kg FD70 suspended in sterile PBS (10 mg/mL; Cat #18912014, Thermo Fisher Scientific). After 4 h, pups were sacrificed and plasma levels of FD70 were measured by fluorometry. The FD70 concentrations in the plasma of each pup were calculated based on a standard curve.

### Tissue collection and injury evaluation

Following abdominal incision, the gastrointestinal tract was carefully removed. Small intestines were evaluated grossly and the terminal 5 cm (ileum) was immediately excised. The terminal 0.5 cm of each sample was placed in buffered 10% paraffin for hematoxylin and eosin (H&E) staining. The severity of mucosal injury was determined on a scale of 0 to 3 by two independent pathologists blinded to H&E staining results. Tissues with histologic scores ≥ 2 were considered as exhibiting NEC 23, 24.

### Immunoblot assay

For immunoblot analysis, mouse ileal tissues were lysed using a total protein extraction kit (Cat #P1250, Applygen, Beijing, China) according to the manufacturer's instructions. Total lysates were resolved by electrophoresis using 4-15% precast polyacrylamide gels (Cat #456-1085, Bio-Rad, Hercules, CA, USA), transferred to polyvinylidene fluoride membranes (Cat #IPVH00010, Millipore, Burlington, VT, USA), and incubated overnight at 4 °C with antibodies against ZO-1 (Cat #ab216880), AMPK (Cat #ab131512), p-AMPK (Cat #ab23875), SIRT1 (Cat #ab12193), Foxp3 (Cat #ab10901), RORγt (Cat #ab207082), STAT3 (Cat #ab68153), p-STAT3 (phospho S727; Cat #ab30647), STAT5 (Cat #ab16276), p-STAT5 (phospho Y694; Cat #ab32364), and β-actin (Cat #ab179467) (all from Abcam, Shanghai, China). The membranes were incubated with horseradish peroxidase (HRP)-conjugated secondary antibodies (goat anti-rabbit IgG-HRP; Cat #ab6721, Abcam) for 1 h at room temperature and detected using enhanced chemiluminescence substrate (Cat #32109, Thermo Fisher Scientific). Immunoblot images were analyzed using Image Lab software (Bio-Rad).

### Preparation of lamina propria mononuclear cells (LPMCs) for flow cytometry

LPMCs were isolated from mouse ileum specimens using the murine Lamina Propria Dissociation Kit (Cat #130-097-410, Miltenyi Biotec, Bergisch-Gladbach, Germany) as previously described 5. Briefly, the specimens were cleaned of mesentery, opened longitudinally, and fragmented with scissors; then, they were incubated in Hank's balanced salt solution (HBSS) without Ca^2+^ and Mg^2+^ containing 10 mM HEPES, 5 mM EDTA, 5% fetal bovine serum (FBS), and 1 mM dithiothreitol for 20 min with continuous shaking at 37 °C. Supernatants containing the intraepithelial lymphocytes were removed. The residual lamina propria was cut into smaller pieces and digested in HBSS with Ca^2+^ and Mg^2+^ containing 10 mM HEPES, 10% FBS, 1 mg/mL collagenase D, 0.1 mg/mL DNAse I, and 0.1 U/mL dispase at 37 °C for 30 min with continuous shaking. LPMCs were washed with PB buffer (PBS with 0.5% bovine serum albumin) and then resuspended in PB buffer for further applications.

### Umbilical cord blood collection and CD4^+^ T cell purification

Cord blood mononuclear cells were isolated from the umbilical cord blood obtained immediately after the delivery of healthy infants by Ficoll-Paque density gradient centrifugation (Cat #17-5442-02, GE Healthcare, Little Chalfont, UK). Then, naïve CD4^+^ T cells (CD4^+^ CD45RA^+^ CD45RO^-^) were negatively selected using the human Naïve CD4^+^ T Cell Isolation Kit II (Cat #130-094-131, Miltenyi Biotec). The purity of isolated CD4^+^ CD45RA^+^ T cells was > 90% as determined by flow cytometric analysis using CD4-FITC and CD45RA-PE staining. Sample collection was performed in accordance with the ethical standards of the Ethics Committee of The Sixth Affiliated Hospital, Sun Yat-sen University (2019ZSLYEC-080) and written informed consent was obtained from all participating pregnant women.

### Lentiviral transduction and RNA interference of SIRT1

Lentiviruses encoding full-length human *SIRT1* cDNA (SIRT1 LV) and those containing shRNA against SIRT1 were designed and synthesized by GENECHEM (Shanghai, China). The shRNA nontarget sequence was 5′- TTCTCCGAACGTGTCACGT-3′ and the SIRT1-targeting sequences were as follows: #1, 5′-CAGGTCAAGGGATGGTATTTA-3′; #2, 5′-CATGAAGTGCCTCAGATATTA-3′; #3, 5′-GCGGCTTGATGGTAATCAGTA-3′. Viruses were produced and titrated in HEK293T cells according to the manufacturer's instructions. Lentiviruses expressing empty plasmids (CTRL LV) and containing nonspecific shRNA (CTRL shRNA) were used as controls.

### *In vitro* CD4^+^ T cell polarization assay

Highly purified CD4^+^ T cells were cultured in TexMACS medium (Cat #130-097-196, Miltenyi Biotec) supplemented with 50 µM 2-mercaptoethanol (Cat #2198502, Thermo Fisher Scientific) and penicillin/streptomycin (Cat #15140122, 100 IU/mL, Invitrogen, Carlsbad, CA, USA). Naïve CD4^+^ T cells were stimulated with anti-CD3/CD28-coated microbeads at a 1:1 bead-to-cell ratio (Cat #11132D, Thermo Fisher Scientific) in the presence of IL-1β (10 ng/mL; Cat #200-01B, Peprotech), IL-6 (20 ng/mL; Cat #200-06, Peprotech), TGF-β (5 ng/mL; Cat #240-B, R&D Systems, Minneapolis, MN, USA), anti-IFN-γ (1 µg/mL; Cat #MAB285, R&D Systems), and anti-IL-4 (1 µg/mL; Cat #MAB204-100, R&D Systems) for Th17 cell polarization 25 or in the presence of IL-2 (100 IU/mL; Cat #200-02, Peprotech) and TGF-β (5 ng/mL; Cat #240-B, R&D Systems) for Treg polarization for 4 days. Melatonin (Cat #S1204, Selleck Chemicals) was added at the start of culture and at day 2, at various concentrations (0, 2, 20, and 200 ng/mL). Vehicle consisting of < 25% ethanol in PBS was used as a control (VEH). For some Th17 cell- and Treg cell-differentiation experiments, Ex-527 (Cat #S1541), SRT1720 (Cat # S1129), or Compound C (Cat #S7840, all purchased from Selleck Chemicals) was added as indicated in the corresponding figures. To confirm the role of SIRT1 in naïve CD4^+^ T cell differentiation towards Th17 or Treg cells, the SIRT knockdown or overexpression was performed by transfecting specific SIRT1 shRNA or SIRT1 LV into naïve CD4^+^ T cells prior to activation. At 24 h after transfection, T cells were stimulated under Th17 or Treg conditions for 4 days.

### Enzyme-linked immunosorbent assay (ELISA) for cytokines and melatonin

The concentrations of cytokines, including IL-10 (Cat #CSB-E04593h), IL-17 (Cat #CSB-E12819h), IL-22 (Cat #CSB-E13418h), and TGF-β (Cat #CSB-E04725h), were measured in the culture supernatants using commercial ELISA kits (all purchased from CUSABIO, Wuhan, China) according to the manufacturer's protocols. Intestinal melatonin was also determined using commercial ELISA kits (Cat #E-EL-M0788c, Elabscience, Wuhan, China) according to the manufacturer's instructions.

### Real-time quantitative reverse transcription-polymerase chain reaction (qRT-PCR)

Total RNA was extracted from cultured CD4^+^ T cell subsets using TRIzol reagent (Cat #15596026, Invitrogen) and reverse transcribed into cDNA with random hexamers using the SuperScript III First-Strand Synthesis system (Cat #18080-044, Invitrogen). cDNA was analyzed using the Fast SYBR Green PCR Master Mix (Cat #4385612, Applied Biosystems, Foster City, CA, USA) in the 7500 real-time PCR system (Applied Biosystems) for the target genes (Supplementary Table 1). The relative expression of the target genes was normalized to that of β-actin and calculated as 2^(Ct(β-actin - gene of interest))^, according to our previous study^5^.

### Flow cytometry

For intracellular cytokine staining, cells (1 × 10^6^/mL) were stimulated at 37 °C for 5 h with the Leukocyte Activation Cocktail (Cat #550583, BD Biosciences, San Jose, CA, USA) in the presence of BD GolgiStop™ Protein Transport Inhibitor (containing Brefeldin A; Cat #554724, BD Biosciences) and then pretreated with Fc block CD16/CD32 antibodies (Cat #553142, BD Biosciences; clone: 2.4G2) to block nonspecific binding. After being stained for surface markers (Supplementary Table 2), cells were fixed and permeabilized using Cytofix/Cytoperm (Cat #555028, BD Biosciences) and then stained with fluorochrome-coupled antibodies against Foxp3 and IL-17A (Supplementary Table 2) according to the manufacturer's instructions. Fluorescence data were collected using a FACS Canto II (BD Biosciences) and then analyzed with FlowJo software (Ashland, OR, USA).

### Statistical analysis

Unless otherwise specified, data are expressed as the mean ± standard deviation (SD) and were analyzed using Prism software version 8.0 (GraphPad, La Jolla, CA, USA) and SPSS software version 21 (IBM, Hampshire, UK). Statistical significance between two groups was analyzed using the Student's t-test. Differences among three or more groups were evaluated using one-way analysis of variance (ANOVA) with a Bonferroni multiple comparison test or Kruskal-Wallis pairwise comparison test. Survival curves were examined using Kaplan-Meier estimates and log-rank tests. A value of *P* ≤ 0.05 was considered statistically significant.

## Results

### Melatonin prevents Th17/Treg imbalances to ameliorate NEC

**Figure 1:**
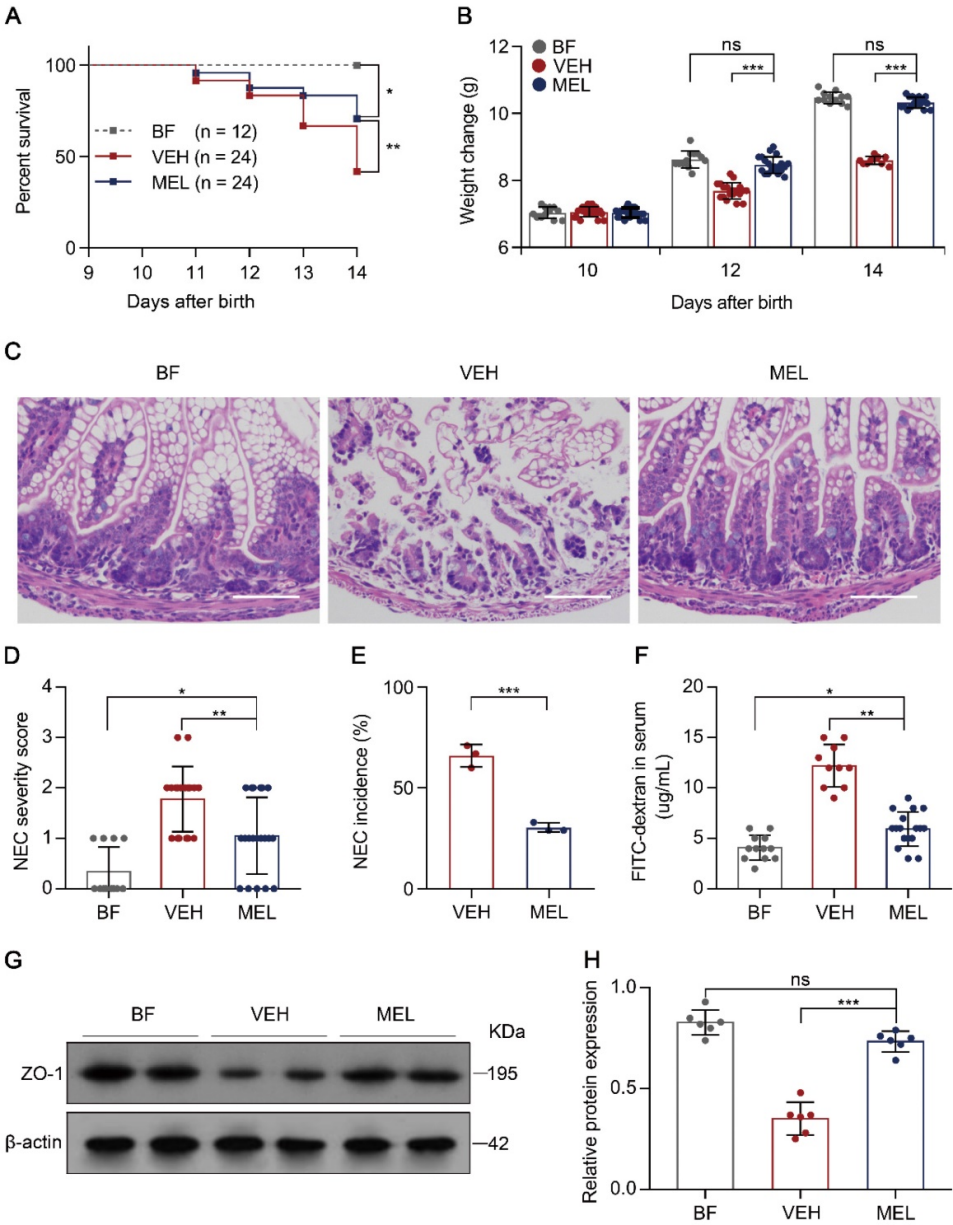
** Therapeutic effect of melatonin in mouse pups on necrotizing enterocolitis (NEC). (A)** Kaplan-Meier estimates and log-rank tests were used to analyze the survival rates of breastfed (BF) and NEC pups upon melatonin treatment (MEL) or treatment with vehicle (VEH). *P < 0.05; **P < 0.01. **(B)** Weight change in mouse pups in BF, VEH, and MEL groups. **(C)** Representative histology (H&E staining) of ileal sections in BF, VEH, and MEL pups. Scale bar, 50 µm. **(D)** Quantification of the severity of **(C)** (n = 12 for BF, 18 for VEH, and 20 for MEL). **(E)** Incidence of NEC (damage scores > 2) in VEH and MEL pups. **(F)** Fluorescence readings in plasma 4 h after gavage with FITC-dextran from BF (n = 12), VEH (n = 10), and MEL (n = 17) groups. **(G)** Immunoblot analysis of the expression of ZO-1 in the ilea of BF, VEH, and MEL pups. **(H)** Quantification of **(G)** (n = 6). Each symbol (**B**, **D**, **F**, **H**) represents an individual mouse and **(E)** shows the incidence of each independent experiment (n = 24); column graphs represent the mean with error bars indicating standard deviation (SD), *P < 0.05; **P < 0.01; ***P < 0.001, ns: not significant. *P* values were derived from one-way ANOVA followed by the Bonferroni multiple comparison test (**B**, **D**, **F**, **H**) or an unpaired two-tailed Student's t-test (**E**). Data are representative of three independent experiments (**A**, **B**, **D**-**F**, **H**).

To determine the protective effect of melatonin on NEC, we first subjected newborn mice to a NEC model characterized by extensive inflammation and necrosis resulting from an imbalance caused by increased Th17 cells and diminished Treg cells 5, 6. As shown in Figure S1, the administration of melatonin during the generation of the mouse model of NEC resulted in a significant reduction in mortality, poor weight gain, disease severity, and morbidity as compared with the presentation in vehicle-injected controls (Figure 1A-E), supporting the mitigating role of melatonin observed in both a rat model and human infants with NEC 19, 20. Furthermore, we evaluated the gut barrier function by administering FITC-dextran via gavage to all surviving pups 4 h prior to sacrifice and measured the concentrations of FITC-dextran in the plasma 26. As depicted in Figure 1F, melatonin administration resulted in a significant reduction of FITC-dextran in the plasma. Consistent with this result, melatonin administration prevented the decrease in zonula occludens-1 (ZO-1) (Figure 1G-H), a biomarker of tight junctions 27.

**Figure 2:**
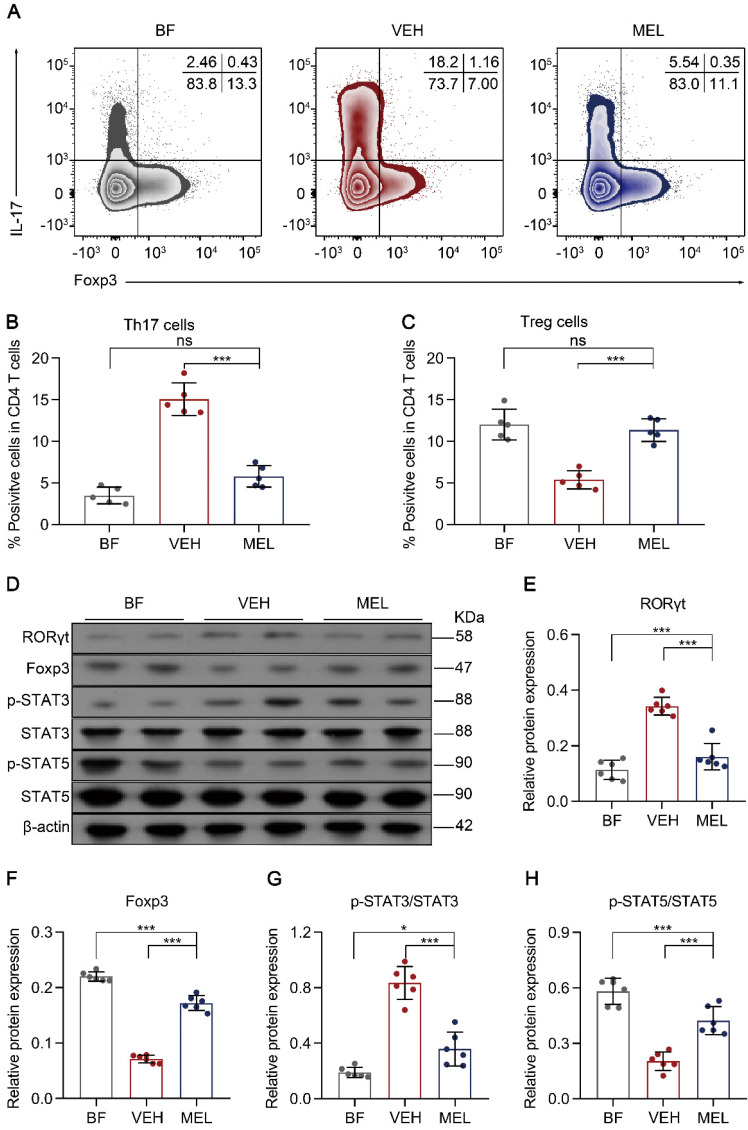
** Melatonin effects are associated with the lamina propria Th17/Treg balance. (A)** Representative flow cytometry plots of RORγt and Foxp3 expression in gated lamina propria CD4^+^ T cells from ileum sections of breastfed (BF) and necrotizing enterocolitis (NEC) model pups upon melatonin treatment (MEL) or treatment with vehicle (VEH). **(B, C)** Quantification of the percentages of Th17 (**B**) and Treg (**C**) cells in (**A**), n = 5 per group. **(D)** Immunoblot analysis of RORγt, Foxp3, p-STAT3, STAT3, p-STAT5, and STAT5 in the ilea of BF, VEH, and MEL pups; β-actin was used as an internal control. (**E-H**) Quantification of RORγt (**E**), Foxp3 (**F**), p-STAT3/STAT3 (**G**), and p-STAT5/STAT5 (**H**), n = 6 per group. Each symbol (**B**, **C**, **E**-**H**) represents an individual mouse; column graphs represent the mean with error bars indicating standard deviation (SD), *P < 0.05; ***P < 0.001; ns: not significant. *P* values were derived through one-way ANOVA followed by the Bonferroni multiple comparison test (**B**, **C**, **E**-**H**). Data are representative of three independent experiments (**B**, **C**, **E**-**H**).

Next, we investigated whether the beneficial effects of melatonin treatment were associated with the balance between Th17 and Treg cells. As shown in Figure 2A-C, we observed a significant reduction of Th17 cells and an increase of Treg cells in the intestinal tissues of mice treated with melatonin as compared to the levels obtained in vehicle-injected controls. Additionally, a similar inverse expression pattern was observed for the key transcriptional factors RORγt and Foxp3 (Figure 2D-F). Furthermore, we noted that the phosphorylation of STAT3 and STAT5, which are important for the respective differentiation of Th17 and Treg cells, was decreased and increased, respectively, in melatonin-treated mice compared to that in vehicle-injected controls (Figure 2D and Figure 2G-F).

We also measured the levels of the Th17- and Treg-related cytokines IL-17, IL-22, TGF-β, and IL-10. Compared to that in vehicle-injected controls, melatonin administration had no effect on *Il10* gene expression (Figure S2D) but significantly increased the mRNA levels of *TGF-β* and decreased those of *IL-17* and *IL-22* in the intestinal tissues (Figure S2A-C). Together, these findings support the contention that melatonin significantly attenuates the pathology of NEC and that this might be due, in part, to recovery of the Th17/Treg balance.

### Melatonin-mediated protective effects are Th17/Treg balance-dependent

**Figure 3:**
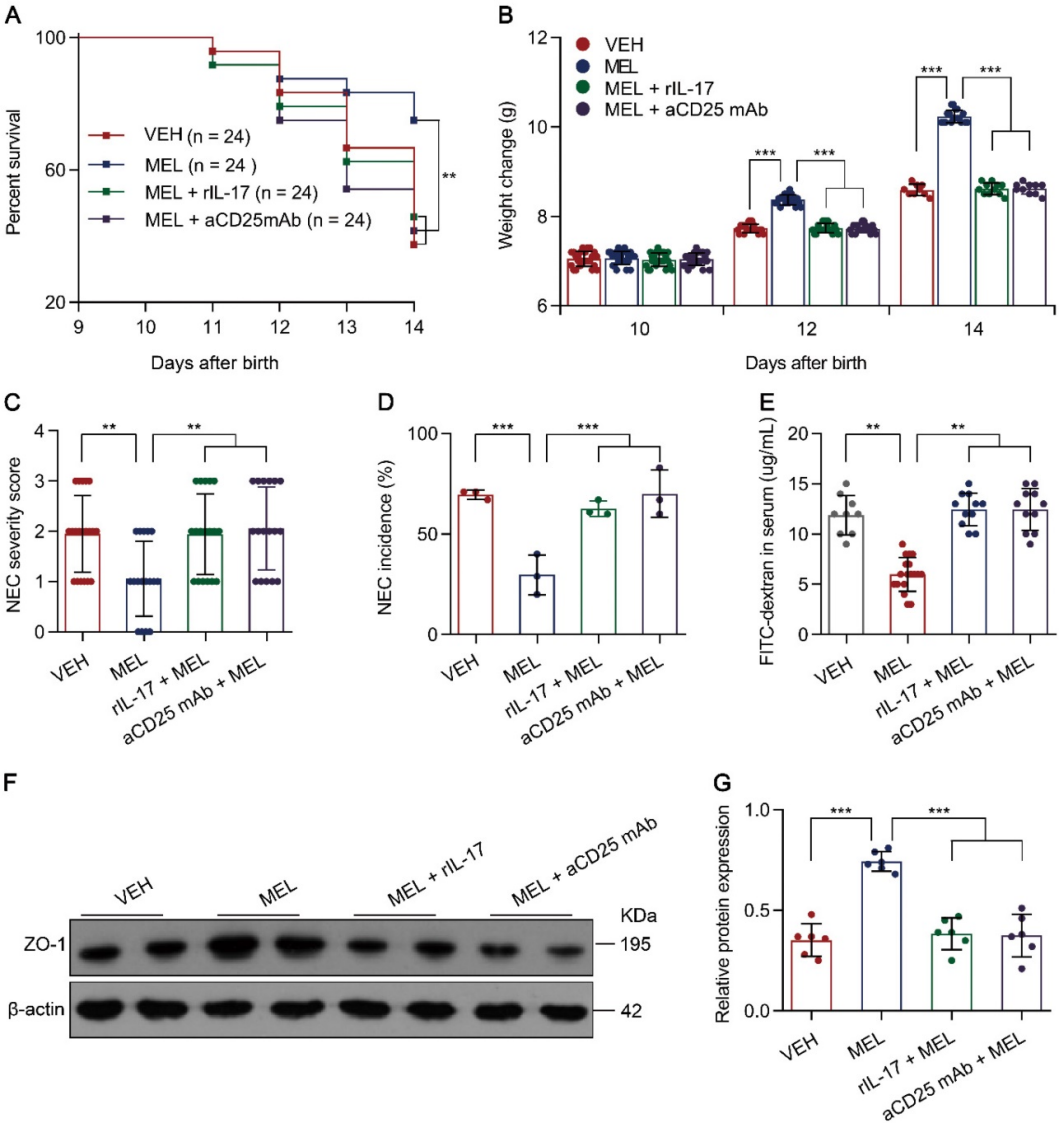
** Melatonin effects require Th17/Treg balance. (A)** Kaplan-Meier estimates and log-rank tests were used to analyze the survival rates of pups following necrotizing enterocolitis (NEC) induction upon treatment with only vehicle (VEH) or with melatonin (MEL), melatonin combined with rIL-17 (MEL + rIL-17), or melatonin combined with aCD25mAb (MEL + aCD25mAb). **P < 0.01.** (B)** Weight change of mouse pups in VEH, MEL, MEL + rIL-17, and MEL + aCD25mAb groups. **(C)** NEC severity scores of the histopathological evaluation of mouse ilea (n = 20 for VEH, 17 for MEL, 18 for MEL + rIL-17, and 17 for MEL + aCD25mAb groups). **(D)** Incidence of NEC (damage scores > 2) in VEH, MEL, MEL + rIL-17, and MEL + aCD25mAb groups. **(E)** Fluorescence readings in plasma 4 h after gavage with FITC-dextran from VEH (n = 9), MEL (n = 18), MEL + rIL-17 (n = 11) and MEL + aCD25mAb (n = 11) groups. **(F)** Immunoblot analysis of the expression of ZO-1 in ilea of VEH, MEL, MEL + rIL-17 and MEL + aCD25mAb group pups. **(G)** Quantification of **(F)** (n = 6). Each symbol (**B**, **C**, **E**, **G**) represents an individual mouse and (**D**) shows the incidence of each independent experiment (n = 24); column graphs represent the mean with error bars indicating standard deviation (SD), **P < 0.01; ***P < 0.001. *P* values were derived through one-way ANOVA followed by the Bonferroni multiple comparison test (**B**-**E**, **G**). Data are representative of three independent experiments (**A**-**E**, **G**).

Considering that the Th17/Treg imbalance is crucial for the development of NEC 5, 6 and melatonin could restore Th17/Treg balances (Figure 2), we therefore hypothesized that the benefit conferred by melatonin is dependent on the Th17/Treg balance. To evaluate the effects of the melatonin-induced decrease in Th17 cells in NEC, recombinant Il-17 (rIL-17) was administered to mouse pups via intraperitoneal injection at the indicated time points (Figure S1). As predicted, rIL-17 administration attenuated the protective effect of melatonin in mice with NEC (Figure 3A-G).

Next, to investigate the role of Treg cells, an anti-CD25 monoclonal antibody (aCD25 mAb) that could partially mediate Treg cell ablation (Figure S3A-D) was employed 28. Similarly, the intraperitoneal injection of aCD25 mAb at the indicated time points (Figure S1) also impaired the protective effect of melatonin in mice with NEC (Figure 3A-G). The impairment of melatonin treatment was comparable between rIL-17 and aCD25 mAb treatments (Figure 3A-G). These results suggested that the benefits conferred by melatonin are dependent on the Th17/Treg balance.

### Melatonin interferes with Th17 and promotes Treg differentiation

**Figure 4:**
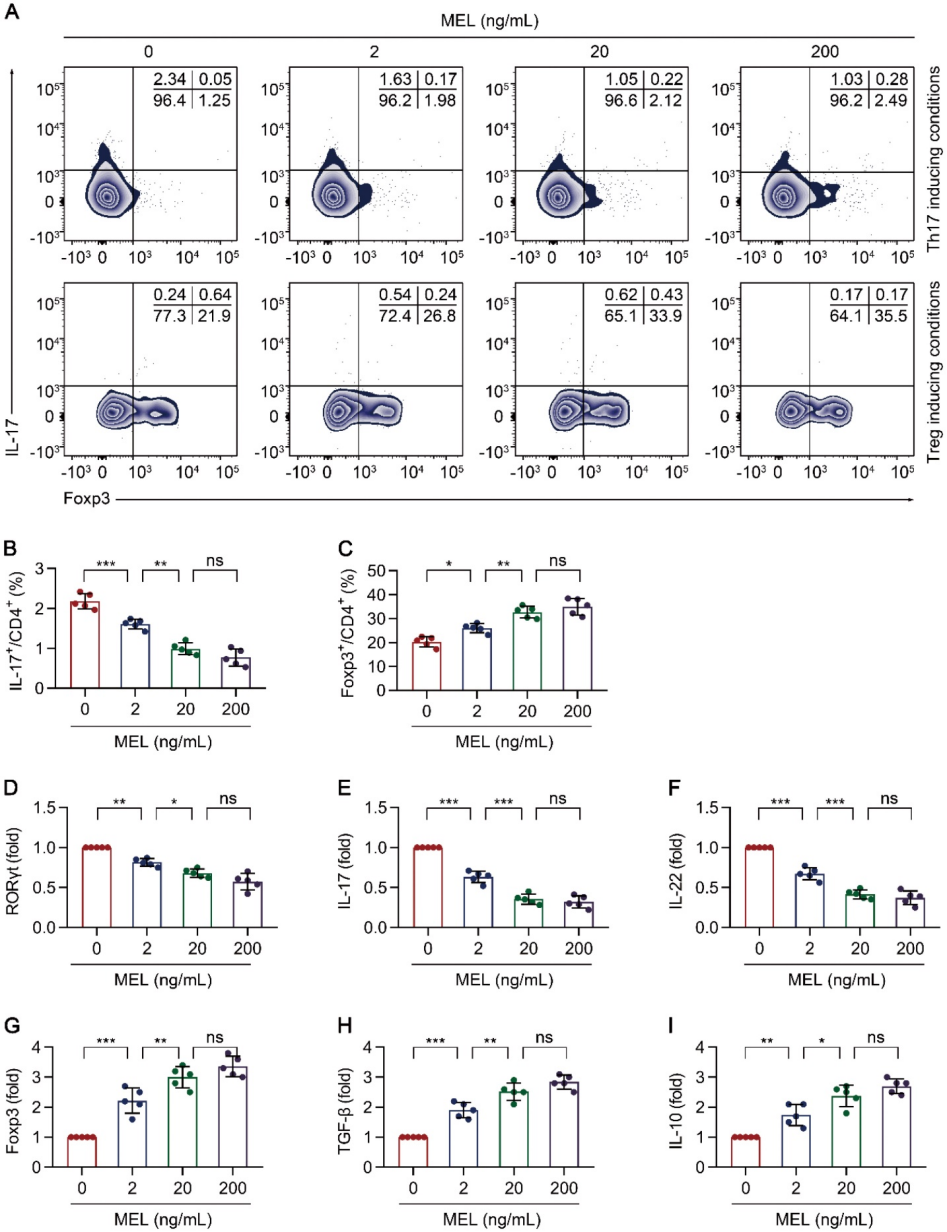
** Melatonin attenuates Th17 and promotes Treg differentiation.** Sorted naïve CD4^+^ T cells (CD3^+^ CD4^+^ CD45RA^+^ CD45RO^-^) of umbilical cord bloods from healthy newborns were differentiated under Th17 or Treg conditions for 5 days, in the presence of melatonin at different concentrations (0 to 200 ng/mL). **(A)** Representative flow cytometric plots of IL-17 and Foxp3 expression in gated CD4^+^ T cells under Th17 (top) and Treg (bottom) conditions. (**B, F**) Quantification of the frequency of Th17 (**B**) and Treg (**F**) cells in (**A**). **C**-**E**, **G**-**I** Real time qRT-PCR analysis of relative mRNA expression of *RORγt* (**C**), *IL-17* (**D**), *IL-22* (**E**), *Foxp3* (**G**), *TGF-β* (**H**), and *IL-10* (**I**) in Th17 (**C**-**E**) and Treg (**G**-**I**) conditions (as described in the Methods). Each symbol (**B**-**I**) represents an individual experiment (n = 5); column graphs represent the mean with error bars indicating standard deviation (SD), *P < 0.05; **P < 0.01; ***P < 0.001; ns: not significant. *P* values were derived through one-way ANOVA followed by the Bonferroni multiple comparison test (**B**-**I**).

To further understand the impact of melatonin on Th17/Treg balance-dependent beneficial effects in NEC (Figure 3), we examined the effect of melatonin on the differentiation of CD4^+^ T cells under Th17 and Treg cell-polarizing conditions. Under Th17-skewing conditions, melatonin decreased the proportion of Th17 cells, (Figure 4A-B) with consistent results observed for the gene expression of *RORγt*, *IL-17*, and *IL-22* (Figure 4C-E). Concurrently, the levels of IL-17 and IL-22 in the culture supernatants were also decreased upon exposure to melatonin (Figure S4A-B). Conversely, melatonin enhanced the differentiation of Treg cells (Figure 4A, F) together with consistent results on the gene expression of *Foxp3*, *IL-10*, and *TGF-β* under Treg-skewing conditions (Figure 4G-I). The levels of IL-10 and TGF-β in the culture supernatants were also increased upon exposure to melatonin (Figure S4C-D). Together, these data suggested that melatonin suppresses Th17 while facilitating Treg cell differentiation, respectively, under Th17 and Treg cell polarizing conditions.

### Melatonin suppresses Th17 and boosts Treg differentiation via SIRT1 activation

We next sought to determine the molecular mechanisms by which melatonin modulates the differentiation of Th17 and Treg cells. Based on emerging findings that melatonin might increase SIRT1 expression 29, 30, which in turn could regulate the differentiation of Th17 and Treg cells 31, 32, we examined the gene expression of SIRT1 in melatonin-treated CD4^+^ T cells under Th17 and Treg-polarizing conditions. Although the Th17-inducing conditions only resulted in an increasing trend of *SIRT1* mRNA in the presence of melatonin at 6 h after stimulation (Figure S5A), the mRNA expression of *SIRT1* was significantly higher in the melatonin-treated group than in vehicle-treated controls under Treg cell-inducing conditions (Figure S5B).

**Figure 5:**
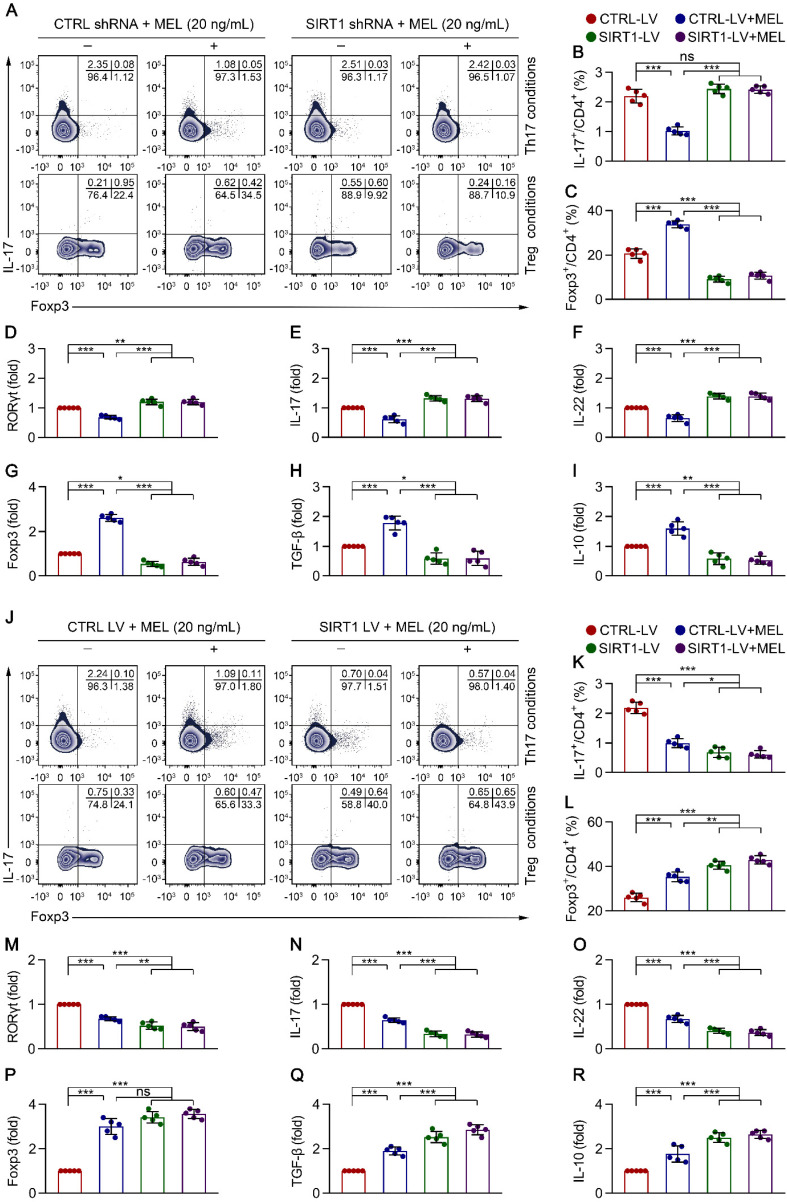
** Melatonin suppresses Th17 and enhances Treg differentiation via SIRT1 activation**. Sorted naïve CD4^+^ T cells (CD3^+^ CD4^+^ CD45RA^+^ CD45RO^-^) from umbilical cord blood of healthy newborns were transduced with lentivirus-containing control-shRNA (CTRL shRNA) and SIRT1-shRNA (SIRT1 shRNA) (**A**-**I**), or control lentivirus (CTRL LV), and SIRT1-expressing lentivirus (SIRT1 LV) (**J**-**R**). At 24 h after transduction, CD4^+^ T cells were differentiated under Th17 or Treg conditions for 4 days in the presence of melatonin (20 ng/mL). (**A**) Representative flow cytometric plots of IL-17 and Foxp3 expression in gated CD4^+^ T cells under Th17 (top) and Treg (bottom) conditions following transduction with CTRL or SIRT1 shRNA. (**B, C**) Quantification of the frequency of Th17 (**B**) and Treg (**C**) cells in (**A**). (**D**-**I**) Real-time qRT-PCR analysis of relative mRNA expression of *RORγt* (**D**), *IL-17* (**E**), *IL-22* (**F**), *Foxp3* (**G**), *TGF-β* (**H**), and *IL-10* (**I**) in Th17 (**D**-**F**) and Treg (**G**-**I**) conditions. **(J)** Representative flow cytometric plots of IL-17 and Foxp3 expression in gated CD4^+^ T cells under Th17 (top) and Treg (bottom) conditions following transduction with CTRL LV or SIRT1 LV. (**K, L**) Quantification the frequency of Th17 (**K**) and Treg (**L**) cells in (**J**). (**M**-**R**) Real-time qRT-PCR analysis relative mRNA expression of *RORγt* (**M**), *IL-17* (**N**), *IL-22* (**O**), *Foxp3* (**P**), *TGF-β* (**Q**), and *IL-10* (**R**) in Th17 (**M**-**O**) and Treg (**P**-**R**) conditions (as described in the Methods). Each symbol (**B**-**I, K**-**R**) represents an individual experiment (n = 5); column graphs represent the mean with error bars indicating standard deviation (SD), *P < 0.05; **P < 0.01; ***P < 0.001; ns: not significant. *P* values were derived from one-way ANOVA followed by the Bonferroni multiple comparison test (**B**-**I, K**-**R**).

To clarify the role of SIRT1 in CD4^+^ T cell differentiation in the presence of melatonin, we performed SIRT1 genetic silencing by transducing naïve CD4^+^ T cells with SIRT1-short hairpin (sh)RNA lentiviral vectors. As shown in Figure 5A-D and Figure 5G, SIRT1 knockdown in CD4^+^ T cells resulted in enhanced Th17 cell differentiation and the mRNA expression of *RORγt*, but the inhibition of Treg cell differentiation and *FOXP3* mRNA expression, in the presence of melatonin. Consistent with these data, SIRT1 deficiency also increased the levels of *IL-17* and *IL-22* but suppressed the expression of *IL-10* and *TGF-β*, as shown by qRT-PCR (Figure 5E-F and Figure 5H-I) and ELISA (Figure S6A). In support of these findings, application of the pharmacologic antagonist Ex-527 also yielded comparable results as observed following SIRT1 knockdown (Figure S7A-C).

To ascertain the role of SIRT1 in CD4^+^ T cell differentiation in the presence of melatonin, we further performed a genetic gain-of-function experiment. Compared to that with the empty vector control, SIRT1 overexpression resulted in decreased Th17 cell differentiation and mRNA expression of *RORγt* but increased Treg cell differentiation and *FOXP3* mRNA expression in the presence of melatonin (Figure 5J-M and Figure 5P). Notably, SIRT1 overexpression also decreased the levels of *IL-17* and *IL-22* but enhanced the production of *IL-10* and *TGF-β*, as shown by qRT-PCR (Figure 5N-O and Figure 5Q-R) and ELISA (Figure S6B). Similarly, application of the SIRT1 activator SRT1720 also yielded similar results as observed following SIRT1 overexpression (Figure S7A-C). These findings suggested that melatonin attenuated Th17 cell- and enhanced Treg cell-differentiation in a SIRT1 activation-dependent manner.

### Blocking the SIRT1 pathway attenuates the protective effects of melatonin in NEC mice

**Figure 6:**
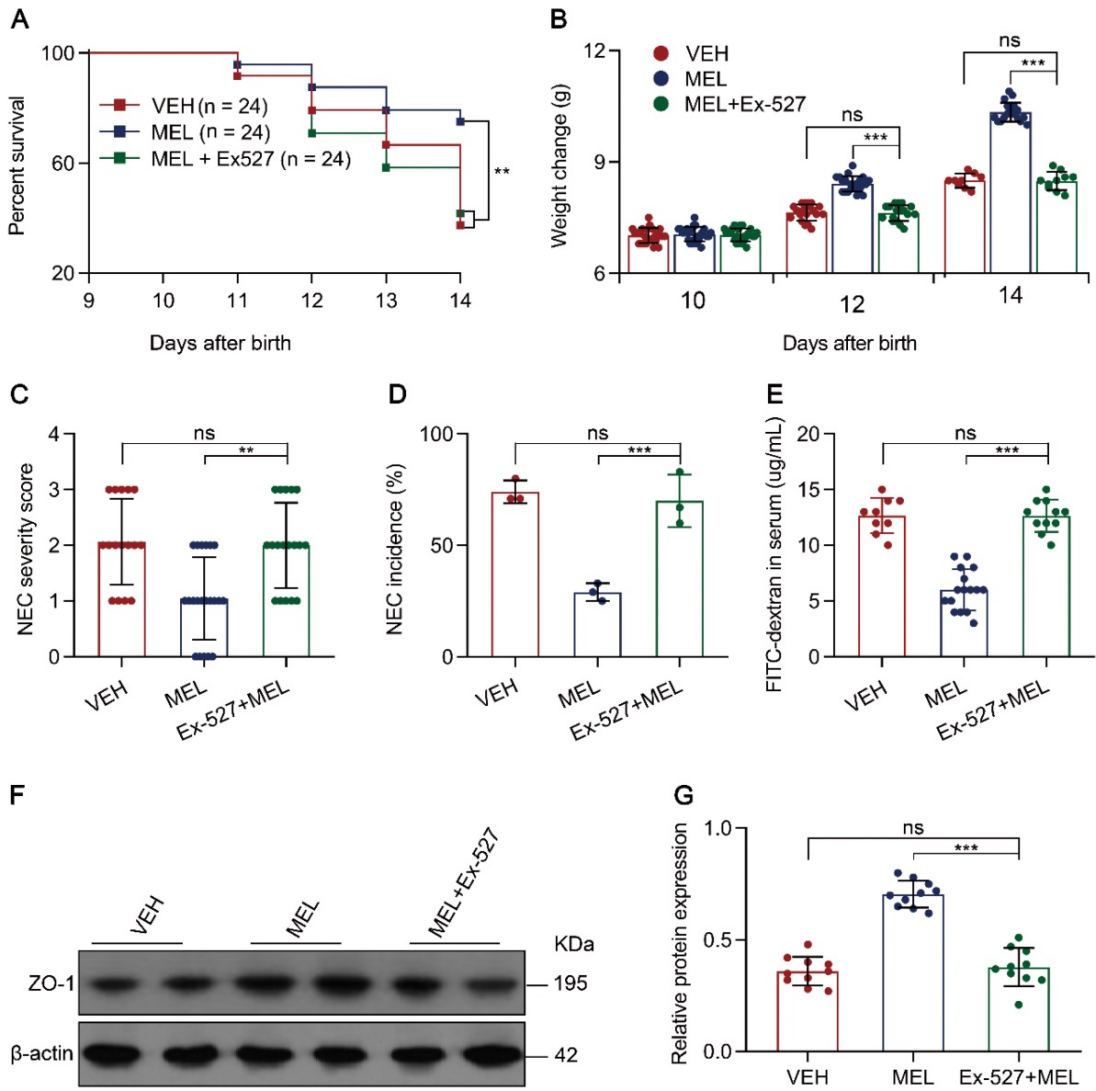
** SIRT1 pathway inhibition attenuates the effects of melatonin. (A)** Kaplan-Meier estimates and log-rank tests were used to analyze the survival rate of pups following necrotizing enterocolitis (NEC) induction upon treatment with only vehicle (VEH) or with melatonin (MEL) or melatonin combined with Ex-527 (MEL + Ex-527). **P < 0.01.** (B)** Weight change of mouse pups in VEH, MEL, and MEL + Ex-527 groups. **(C)** NEC severity scores of the histopathological evaluation of mouse ilea (n = 16 for VEH, 21 for MEL, and 18 for MEL + Ex-527 groups). **(D)** Incidence of NEC (damage scores > 2) in VEH, MEL, and MEL + Ex-527 groups. **(E)** Fluorescence readings in plasma 4 h after gavage with FITC-dextran from VEH (n = 9), MEL (n = 16), and MEL + Ex-527 (n = 11) groups. **(F)** Immunoblot analysis of the expression of ZO-1 in ileum sections of VEH, MEL, and MEL + Ex-527 pups. **(G)** Quantification of **(F)** (n = 6). Each symbol (**B**, **C**, **E**, **G**) represents an individual mouse and (**D**) shows the incidence of each independent experiment (n = 24); column graphs represent the mean with error bars indicating standard deviation (SD), **P < 0.01; ***P < 0.001; ns: not significant. *P* values were derived from one-way ANOVA followed by the Bonferroni multiple comparison test (**B**-**E**, **G**). Data are representative of three independent experiments (**B**-**E**, **G**).

To investigate whether the effects of melatonin occur through SIRT1 activation in vivo, we evaluated the efficacy of the SIRT1 antagonist Ex-527 in melatonin-treated NEC pups (Figure S1). Treatment of recipients with Ex-527 resulted in increased mortality, severity, and morbidity compared with the results of melatonin-treated pups (Figure 6A-D). As expected, the SIRT1 antagonist enhanced the level of FITC-dextran in the plasma compared to that in the melatonin-treated group (Figure 6E). Using immunoblotting, we validated that SIRT1 antagonist administration reduced ZO-1 expression compared to that in melatonin-treated pups (Figure 6F-G). Furthermore, we assessed the inhibitory effects of Ex-527 on intestinal melatonin secretion, and found that there were no effects on intestinal melatonin concentrations in mice with NEC or those breastfeeding (Figure S8). Taken together, these results indicated that the pharmacological blockade of SIRT1 with Ex-527 impairs the therapeutic benefit conferred by melatonin in experimental NEC.

### Blocking the SIRT1 pathway impairs melatonin-mediated improvement of the Th17/Treg balance

**Figure 7:**
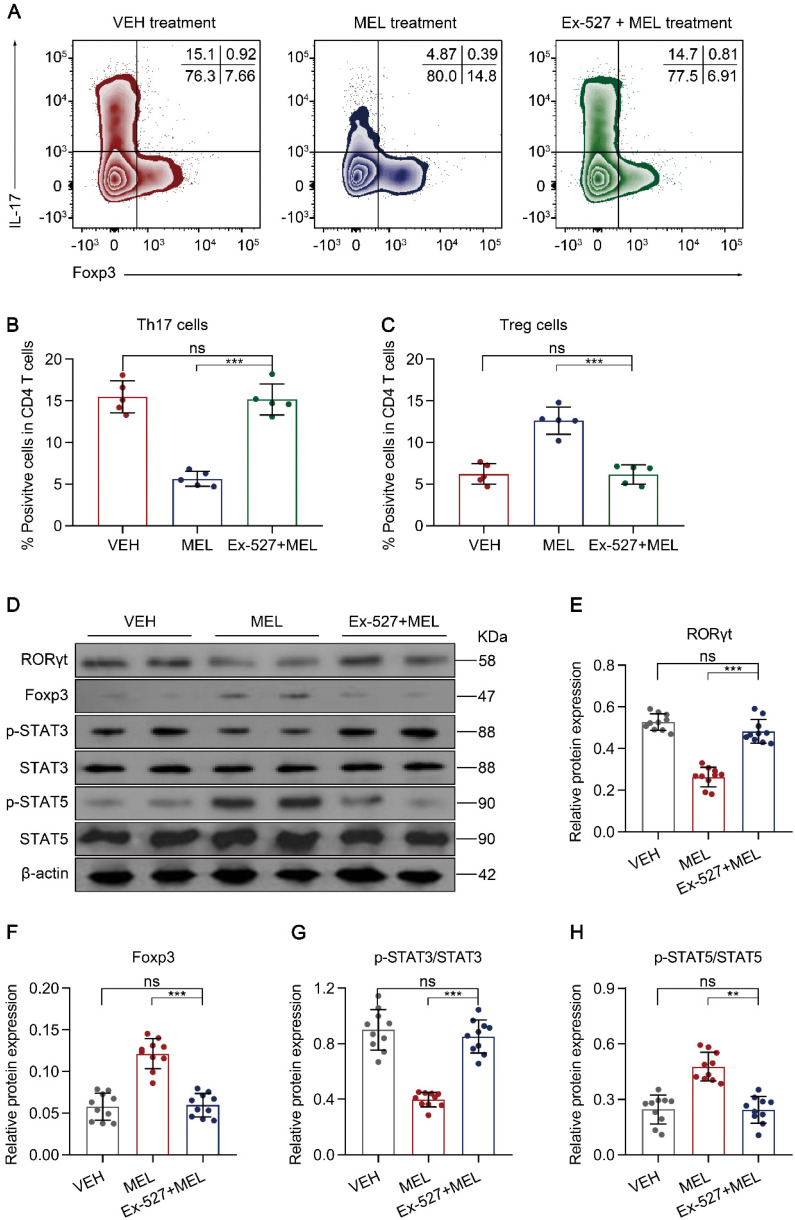
** SIRT1 pathway inhibition attenuates maintenance of the Th17/Treg balance. (A)** Representative flow cytometric plots of IL-17 and Foxp3 expression in gated lamina propria CD4^+^ T cells from ileum sections of pups following necrotizing enterocolitis (NEC) induction upon treatment with vehicle (VEH) alone or with melatonin (MEL) or melatonin combined with Ex-527 (MEL + Ex-527). **(B, C)** Quantification of the percentages of Th17 (**B**) and Treg (**C**) cells in **(A)**, n = 5 per group. **(D)** Immunoblot analysis of RORγt, Foxp3, p-STAT3, STAT3, p-STAT5, and STAT5 in ilea of VEH, MEL, and MEL + Ex-527 pups, β-actin was used as an internal control. (**E-H**) Quantification of RORγt (**E**), Foxp3 (**F**), p-STAT3/STAT3 (**G**), and p-STAT5/STAT5 (**H**). n = 6 per group. Each symbol (**B**, **C**, **E**-**H**) represents an individual mouse; column graphs represent the mean with error bars indicating standard deviation (SD), *P < 0.05; ***P < 0.001; ns: not significant. *P* values were derived from one-way ANOVA followed by a Bonferroni multiple comparison test (**B**, **C**, **E**-**H**). Data are representative of three independent experiments (**B**, **C**, **E**-**H**).

Next, we sought to identify the cellular inflammatory response through which blocking SIRT1 activation impaired the curative effects of melatonin. As melatonin-mediated altered Th17 and Treg differentiation occurred through SIRT1 activation in vitro (Figure 5), we evaluated whether the proportion of Th17 and Treg cells isolated from intestinal tissues was influenced by the SIRT1 antagonist Ex-527. Compared with the results in the melatonin-treated group, the administration of Ex-527 suppressed the Th17 cell decrease along with the Treg cell increase, as indicated by flow cytometry (Figure 7A-C) and immunoblotting (Figure 7D-F). Consistent with these results, Ex-527 also increased STAT3 phosphorylation but decreased STAT5 phosphorylation compared that in melatonin-treated pups (Figure 7D and Figure 7G-H). Moreover, Ex-527 administration also attenuated the production of TGF-β albeit enhanced the levels of IL-17 and IL-22 compared to those following melatonin treatment (Figure S9A-C). Together, these data indicated that Ex-527 impaired the therapeutic effect conferred by melatonin by interfering with the Th17/Treg balance in experimental NEC.

### Melatonin regulates SIRT1 via the AMPK pathway

**Figure 8:**
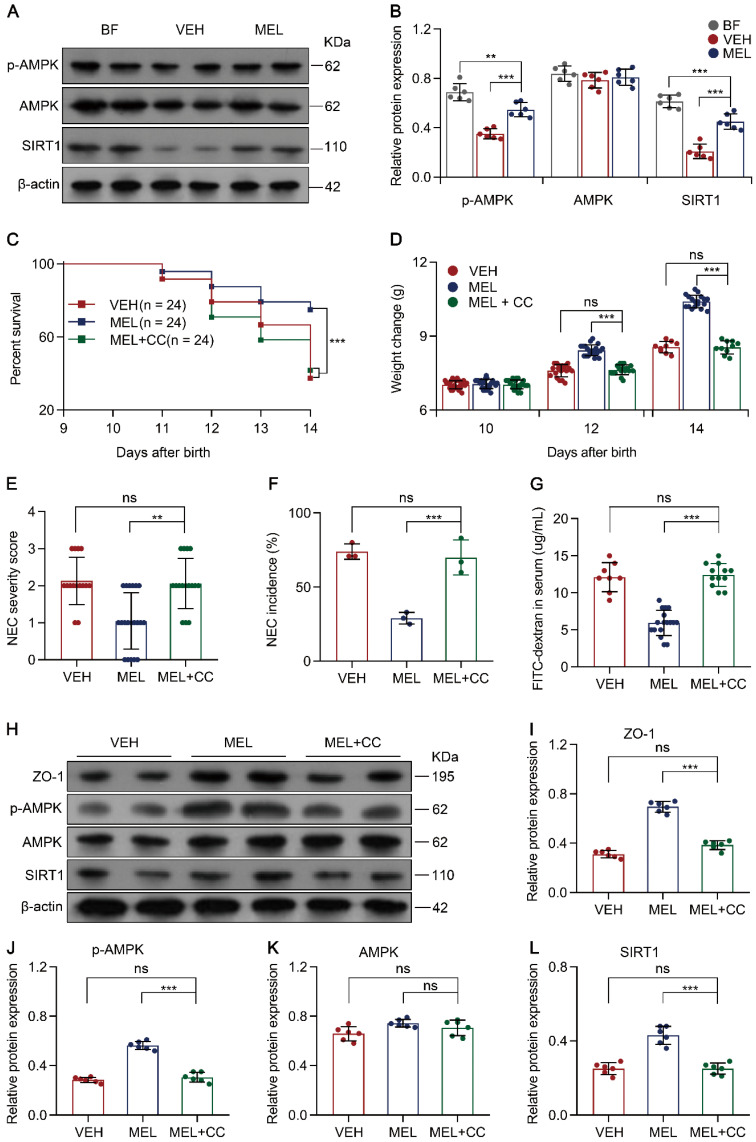
** Melatonin facilitates SIRT1 expression by activating the AMPK pathway. (A)** Immunoblot analysis of p-AMPK, AMPK, and SIRT1 in ileum sections of breastfed (BF) and NEC pups upon melatonin treatment (MEL) or treatment with vehicle (VEH); β-actin was used as an internal control. **(B)** Quantification of p-AMPK, AMPK, and SIRT1 in (**A**), n = 6 per group. **(C)** Kaplan-Meier estimates and log-rank tests were used to analyze the survival rate of pups following NEC induction upon treatment with vehicle (VEH) alone or with melatonin (MEL), or melatonin combined with Compound C (MEL + CC). ***P < 0.001.** (D)** Weight change of mouse pups in VEH, MEL, and MEL + CC groups. **(E)** Necrotizing enterocolitis (NEC) severity scores of the histopathological evaluation of mouse ilea (n = 15 for VEH, 20 for MEL, and 16 for MEL + CC groups). **(F)** Incidence of NEC (damage scores > 2) in VEH, MEL, and MEL + CC groups. **(G)** Fluorescence readings in plasma 4 h after gavage with FITC-dextran from VEH (n = 8), MEL (n = 17), and MEL +CC (n = 12) groups. **(H)** Immunoblot analysis of the expression of ZO-1, p-AMPK, AMPK, and SIRT1 in ileum sections of VEH, MEL, and MEL + CC pups. **(I**-**L)** Quantification of **(H)** (n = 6). Each symbol (**B**, **D**, **E**, **G, I**-**L**) represents an individual mouse and (**f**) shows the incidence of each independent experiment (n = 24); column graphs represent the mean with error bars indicating standard deviation (SD), **P < 0.05; ***P < 0.001; ns: not significant. *P* values were derived through one-way ANOVA followed by the Bonferroni multiple comparison test (**B**-**G**, **I**-**L**). Data are representative of three independent experiments (**D**-**G**, **I**-**L**).

To examine how melatonin affects the expression of SIRT1, the AMPK signaling pathway was evaluated because previous studies have identified AMPK as an upstream mediator of SIRT1 33, 34. Immunoblotting analysis demonstrated that p-AMPK and SIRT1 were all decreased in NEC pups compared to the levels in breastfeed groups (Figure 8A-B). Notably, melatonin treatment upregulated the levels of p-AMPK and SIRT1 in NEC mice (Figure 8A-B). To further assess the function of AMPK in vivo, we used Compound C (a potent AMPK inhibitor 35) to test whether AMPK activation is beneficial for melatonin treatment. As shown in Figure 8C-G, the administration of Compound C in the melatonin-treated mouse model of NEC could abolish the beneficial effects of melatonin treatment. Compound C also caused a decline in ZO-1, p-AMPK, and SIRT1 expression compared to that in melatonin-treated pups (Figure 8H-J and Figure 8L). Similar to Ex-527, Compound C also exerted effects on intestinal melatonin secretion (Figure S8).

**Figure 9:**
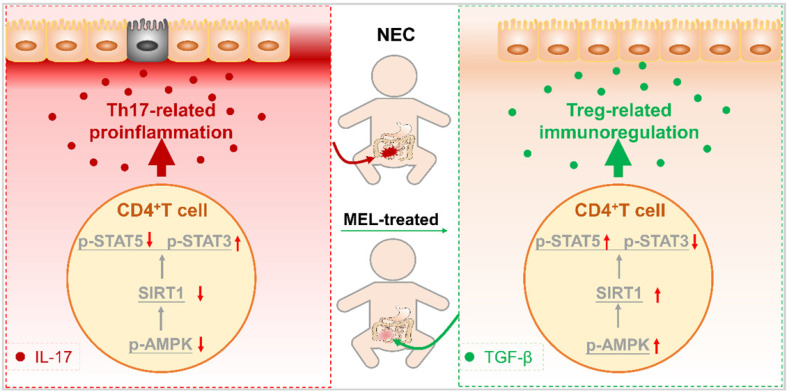
** Schematic of our mechanistic model.** Schematic model illustrating the therapeutic effect of melatonin in a neonatal mouse model of necrotizing enterocolitis. The therapeutic effect of melatonin is Th17/Treg balance-dependent. Melatonin blocks Th17 cell- and promotes Treg cell-differentiation. Melatonin augments AMPK/SIRT1 signaling to promote Treg cell-differentiation.

To confirm that AMPK is also involved in the Th17/Treg balance, the percentage of Th17/Treg cells was analyzed. As shown in Figure S10A-C, Compound C administration prevented the maintenance of Th17/Treg cell balances observed in mice treated with melatonin. Moreover, Compound C administration also enhanced the production of IL-17 and IL-22 but attenuated the levels of TGF-β and IL-10 (Figure S10D-G). Similarly, the addition of Compound C also abolished melatonin-enhanced Treg cell differentiation and attenuated Th17 cell differentiation in vitro (Figure S11A-C). Overall, these results illustrated that AMPK, activated by melatonin, contributes to SIRT1 activation and Th17/Treg balances (Figure 9).

## Discussion

Recent studies indicate that an imbalance caused by diminished Treg cells and increased Th17 cells is a crucial factor in NEC 5-7, 36. In the current study, we demonstrated that melatonin could prevent the imbalance of intestinal Th17/Treg cells in NEC mice. Specifically, we found that melatonin supplementation could ameliorate NEC in a manner dependent on maintaining the intestinal Th17/Treg balance in vivo. In addition, we showed that melatonin inhibited the development of Th17 cells and reciprocally promoted the induction of Treg cells in vitro. Furthermore, mechanistic studies revealed that the balance between Th17 and Treg cells is manipulated by melatonin through activation of the AMPK/SIRT1 pathway. Taken together, these findings clarified that the mechanism by which melatonin exerts a preventive effect in NEC relies upon improving the intestinal Th17/Treg balance (Figure 9).

Melatonin plays a critical role in the prevention of several inflammation-associated neonatal diseases 37-39. Moreover, in newborn rats of NEC models, supplementation with melatonin after disease onset could alleviate intestinal injuries 20. Injection of melatonin as an adjuvant therapy is also associated with improvements in clinical and laboratory outcomes in patients with NEC 19. Increased antioxidant enzyme activities and reduced oxidative stress are considered the major mechanisms through which melatonin ameliorates NEC 20. Notably, our identification of improved intestinal Th17/Treg balance thus represents another mechanism through which melatonin might ameliorate NEC.

Several previous reports have suggested that melatonin could influence the differentiation of Th17 and Treg cells 17. In EAE and hamster models of cholangiocarcinoma, melatonin could decrease IL-17 expression and Th17 infiltration 40-42. Conversely, melatonin treatment could augment the frequency of Treg cells in the peripheral blood of patients with systemic lupus erythematosus and in the central nervous system of EAE models 43, 44. Moreover, we demonstrated in the present study that melatonin facilitates Treg but suppresses Th17 cell differentiation in vitro and in vivo. However, in a murine foregastric carcinoma cell line or metastatic solid tumors, melatonin was found to reduce the number of Treg cells and expression of Foxp3 in the tumor tissues of both humans and mice 45, 46. Such controversial findings indicate that melatonin might inhibit the function of Treg cells under immunosuppressive conditions albeit enhance the number of Treg cells and decrease the number of Th17 cells upon exacerbated immune responses such as those occurring in NEC.

SIRT1 is associated with the differentiation of CD4^+^ T cell subsets and their functions 47. Initial studies indicated that global SIRT1 KO mice were unable to maintain T cell tolerance and developed severe EAE 8, 15. However, recent work using mice with conditional SIRT1 KO in CD4 T cells demonstrated that targeting SIRT1 in T cells significantly reduces Th17 and promote Treg cell differentiation, in turn attenuating EAE or aGVHD 11, 48. In contrast, we showed that Th17 reductions and Treg cell differentiation could result from SIRT1 overexpression in vitro. Our observation corroborates a recent study that reported decreased SIRT1 in Th17-skewing conditions and increased SIRT1 in Treg-polarizing conditions 49. Such discrepancies could result from different microenvironments of T cell differentiation.

Melatonin, as a positive regulator, has been implicated in the modulation of SIRT1 50. We observed that blocking SIRT1 could attenuate the effects of melatonin on Th17 and Treg cell differentiation in vivo. Furthermore, we demonstrated that the AMPK pathway is involved in melatonin-mediated SIRT1 regulation. In comparison, melatonin was shown to activate the AMPK pathway to reduce neuroinflammation and brain damage in a model of traumatic brain injury 51. Melatonin could also inhibit endoplasmic reticulum stress-associated thioredoxin-interacting protein/NLRP3 inflammasome activation through the modulation of AMPK activation in lipopolysaccharide-induced endometritis 52. Additionally, in skeletal muscle, AMPK is involved in energy metabolism through the action of SIRT1 53. Consistent with these studies, our data clearly demonstrated that AMPK, in response to melatonin signaling, contributes to the expression and stabilization of SIRT1, ultimately attenuating intestinal inflammation through the regulation of Th17/Treg balances.

The current study has several limitations. We did not utilize conditional KO mice to clarify the role of SIRT1 in CD4^+^ T cells in the presence of melatonin. Moreover, as AMPK can act both as an upstream and downstream mediator of SIRT1 34, 54, it remains to be determined whether SIRT1 exerts feedback on the AMPK pathway in an NEC background.

## Conclusions

In conclusion, we provide concrete evidence that downregulated AMPK/SIRT1 signaling promotes Th17/Treg imbalances in NEC and that targeting the AMPK/SIRT1 pathway, such as via melatonin, could serve as a potential therapeutic target to modulate NEC pathogenesis.

## Supplementary Material

Supplementary figures and tables.

## Abbreviations

NEC: necrotizing enterocolitis
aCD25 mAb: anti-CD25 monoclonal antibody
RORγt: orphan nuclear receptor
SIRT1: sirtuin 1
KO: knockout
EAE: experimental autoimmune encephalomyelitis
aGVHD: acute graft-versus-host disease
ZO-1: zonula occludens-1
rIL-17: recombinant IL-17
shRNA: short hairpin RNA
qRT-PCR: quantitative reverse transcription-polymerase chain reaction
ELISA: enzyme linked immunosorbent assay
Th17: interleukin-17 (IL-17)-producing CD4^+^ T cell
Treg: Foxp3^+^ regulatory T cell
