# Supplementary material for: Melatonin ameliorates necrotizing enterocolitis by preventing Th17/Treg imbalance through activation of the AMPK/SIRT1 pathway
Source: Theranostics. 2020 Jun 19;10(17):7730–46. doi: 10.7150/thno.45862 (PMC7359097; doi:10.7150/thno.45862)
Supplement: Supplementary file 1 — Supplementary figures and tables. [file thnov10p7730s1.pdf]

Supplementary Information

Melatonin ameliorates necrotizing enterocolitis by preventing Th17/Treg imbalance through activation of the AMPK/SIRT1 pathway

Ma et al.

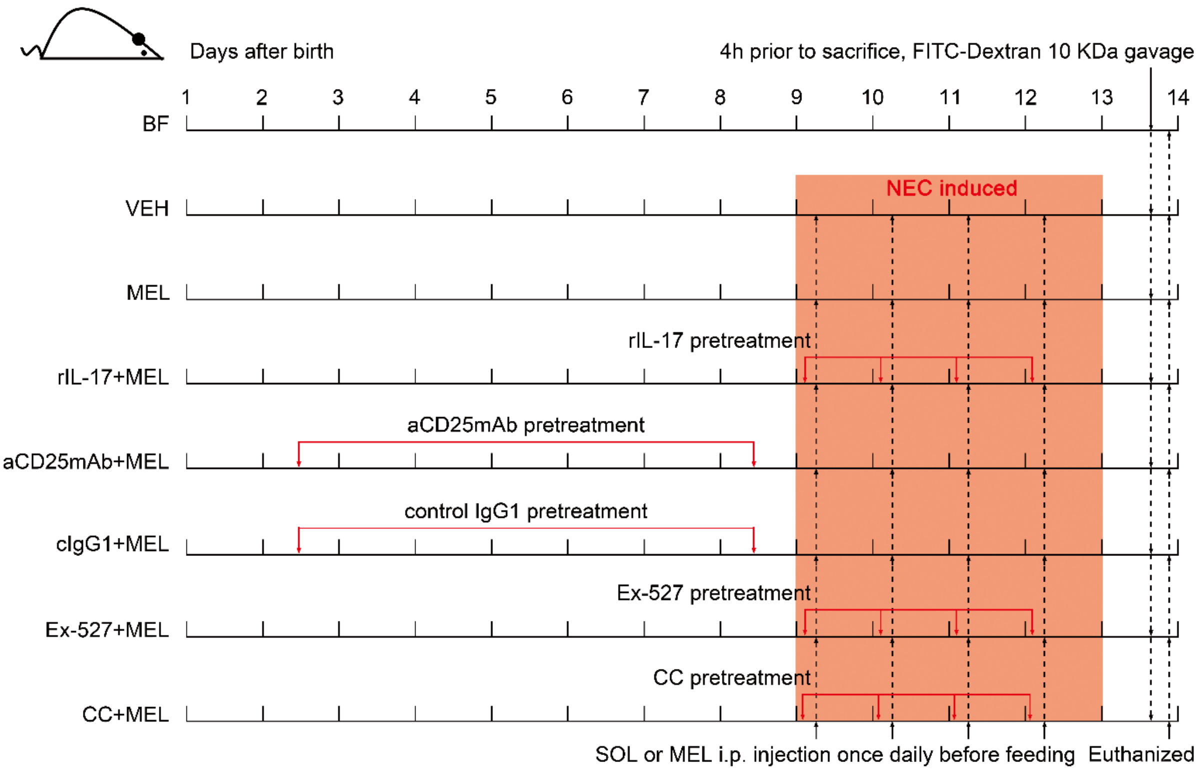

Supplementary Figure 1. Diagrammatic representation of the experimental models. Schematic shows the specific role of melatonin in a mouse necrotizing enterocolitis (NEC) model (melatonin group, MEL), melatonin combined with rIL-17 (melatonin + rIL-17 group, MEL + rIL-17), melatonin combined with aCD25 mAb (melatonin+ aCD25 mAb group, MEL+ aCD25 mAb), melatonin combined Ex-527 (melatonin + Ex-527 group, MEL+ Ex-527), and melatonin combined Compound C (melatonin + Compound C group, MEL+ CC). Naïve control mice (BF group) were observed over the study period without intervention and left with their dams to breastfeed. Simultaneously, controls also included the experimental pups, which were treated only with a vehicle (consisting of < 25% ethanol in PBS, VEH group) or melatonin combined with control IgG1 (melatonin + cIgG1 group, MEL+ cIgG1) at the indicated time points.

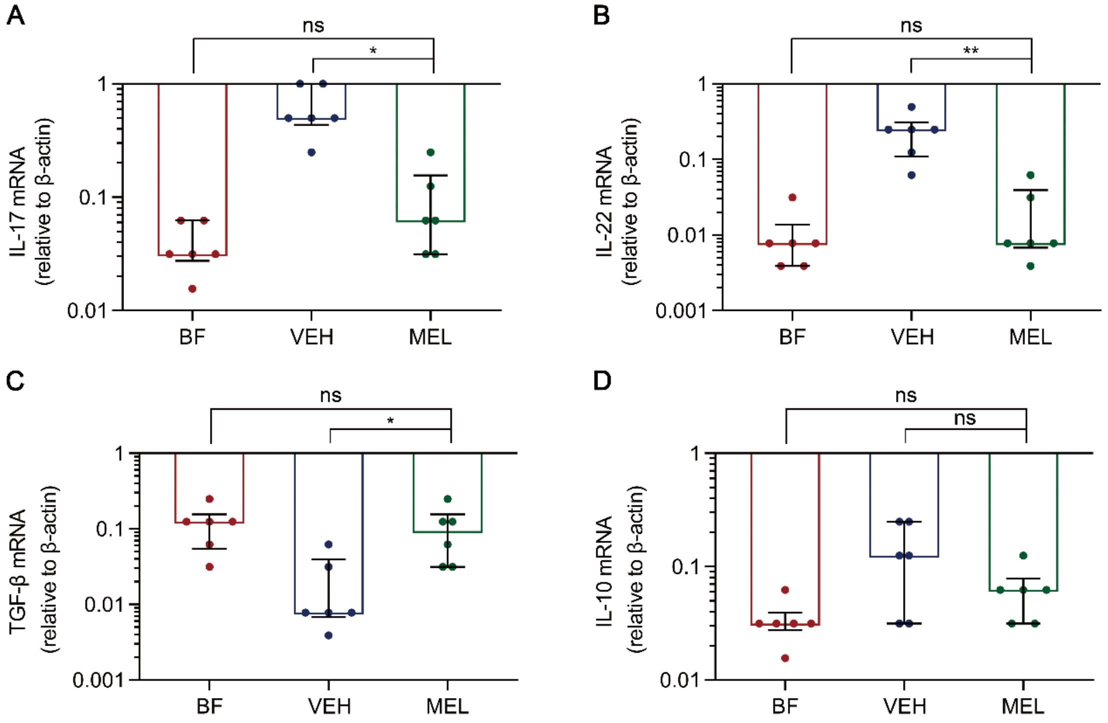

Supplementary Figure 2. (**A–D**) Real-time qRT-PCR analysis of relative mRNA expression of *IL-17* (**A**), *IL-22* (**B**), *TGF-β* (**C**) and *IL-10* (**D**) in ileum of breastfed (BF group) and NEC pups upon melatonin treatment (MEL group) or treatment with vehicle (consisting of < 25% ethanol in PBS, VEH group). Each symbol (**A–D**) represents an individual experiment (n = 6); column graphs represent the median with interquartile range, **P* < 0.05; ***P* < 0.01; ns: not significant. *P* values were calculated using Kruskal-Wallis followed by the Pairwise Comparison test (**A–D**).

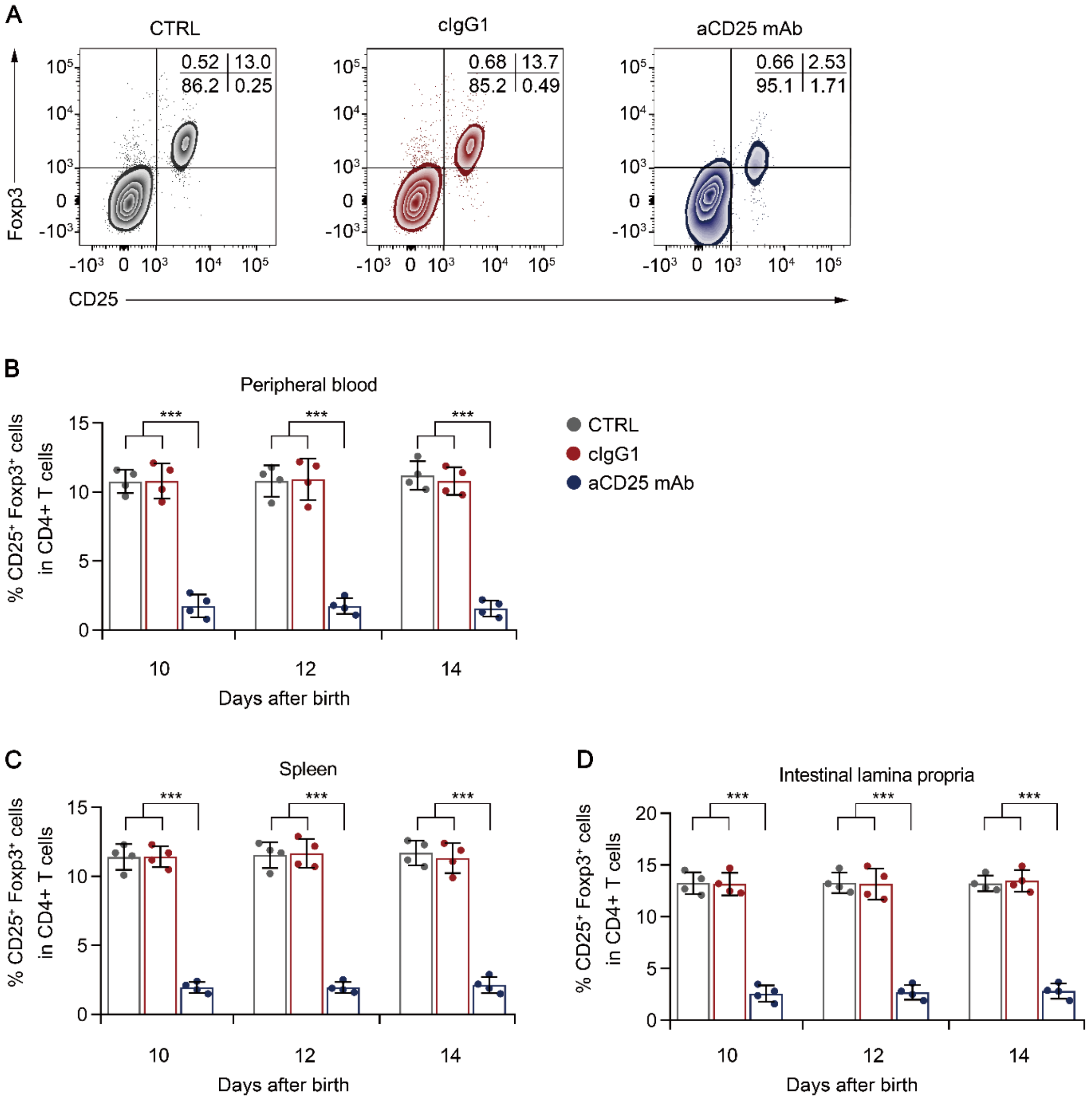

Supplementary Figure 3. Anti-CD25 monoclonal antibody (aCD25 mAb) depletes CD4$^+$ CD25$^+$ Foxp3$^+$ Treg cells. **(A)** Representative flow cytometry plots of CD25 and Foxp3 expression in gated peripheral blood CD4$^+$ T cells from breastfed (CTRL group) and pups upon aCD25 mAb treatment (aCD25 mAb group) or treatment with control IgG1 antibody (cIgG1 group) on day 2 and 8 after birth. **(B–D)** Quantification of the percentages of Treg cells in the peripheral blood (**B**), spleen (**C**) and intestinal lamina propria (**D**) on day 10, 12,

and 14 after birth. Each symbol (**B**–**D**) represents an individual mouse (n = 4); column graphs represent the mean with error bars indicating standard deviation (SD), ***$P < 0.001$. P values were derived through one-way ANOVA followed by the Bonferroni multiple comparison test (**B**–**D**). Data are representative of two independent experiments (**B**–**D**).

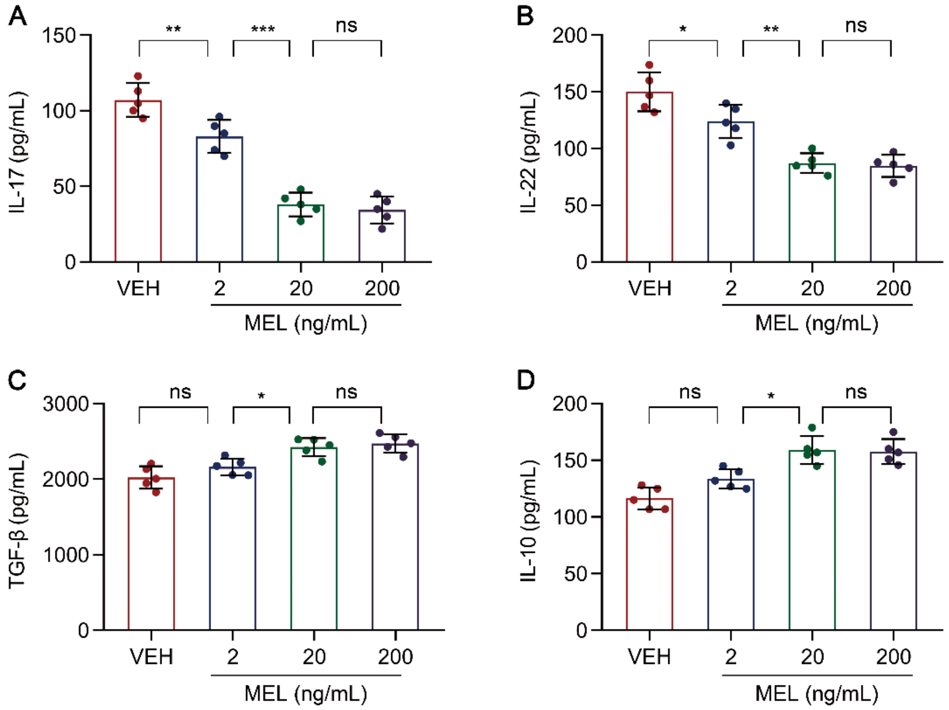

Supplementary Figure 4. (**A**–**D**) The concentrations of IL-17 (**A**), IL-22 (**B**), TGF-β (**C**), and IL-10 (**D**) in the culture supernatants of Th17 (**A, B**) and Treg (**C, D**) conditions, in the presence of melatonin at different concentrations (0 to 200 ng/mL) (as described in Methods). Each symbol (**A**–**D**) represents an individual experiment (n = 5); column graphs represent the mean with error bars indicating standard deviation (SD), $^{*}P < 0.05$; $^{**}P < 0.01$; $^{***}P < 0.001$; ns: not significant. P values were derived through one-way ANOVA followed by the Bonferroni multiple comparison test (**A**–**D**).

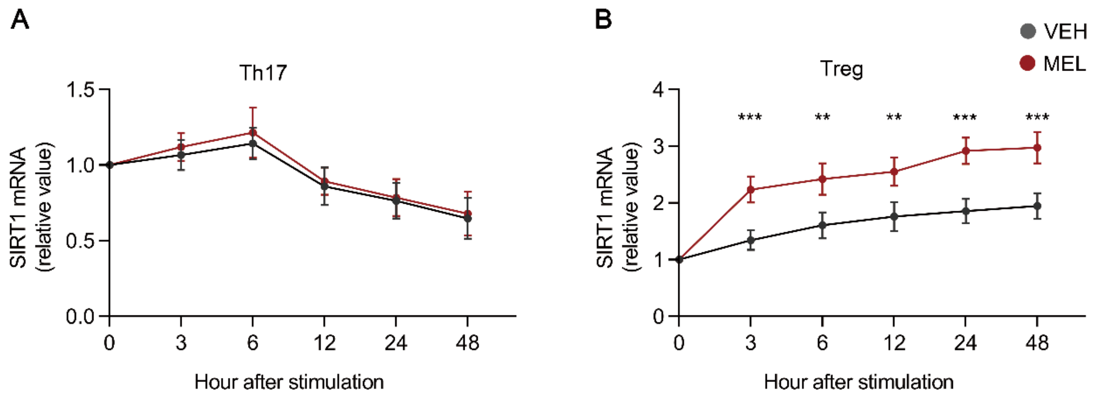

Supplementary Figure 5. *SIRT1* mRNA expression as examined by Real-time qRT-PCR analysis in naïve $CD4^+$ T cells of umbilical cord blood from healthy newborns were activated under Th17 (**A**) or Treg (**B**) conditions for different time courses, in the presence of melatonin (20 ng/ml) (MEL group) or vehicle (consisting of < 25% ethanol in PBS, VEH group). Data are shown as the mean with error bars indicating standard deviation (SD), n = 4. **P<0.01 and ***P<0.001. *P* values were calculated using the nonparametric Student's *t*-tests.

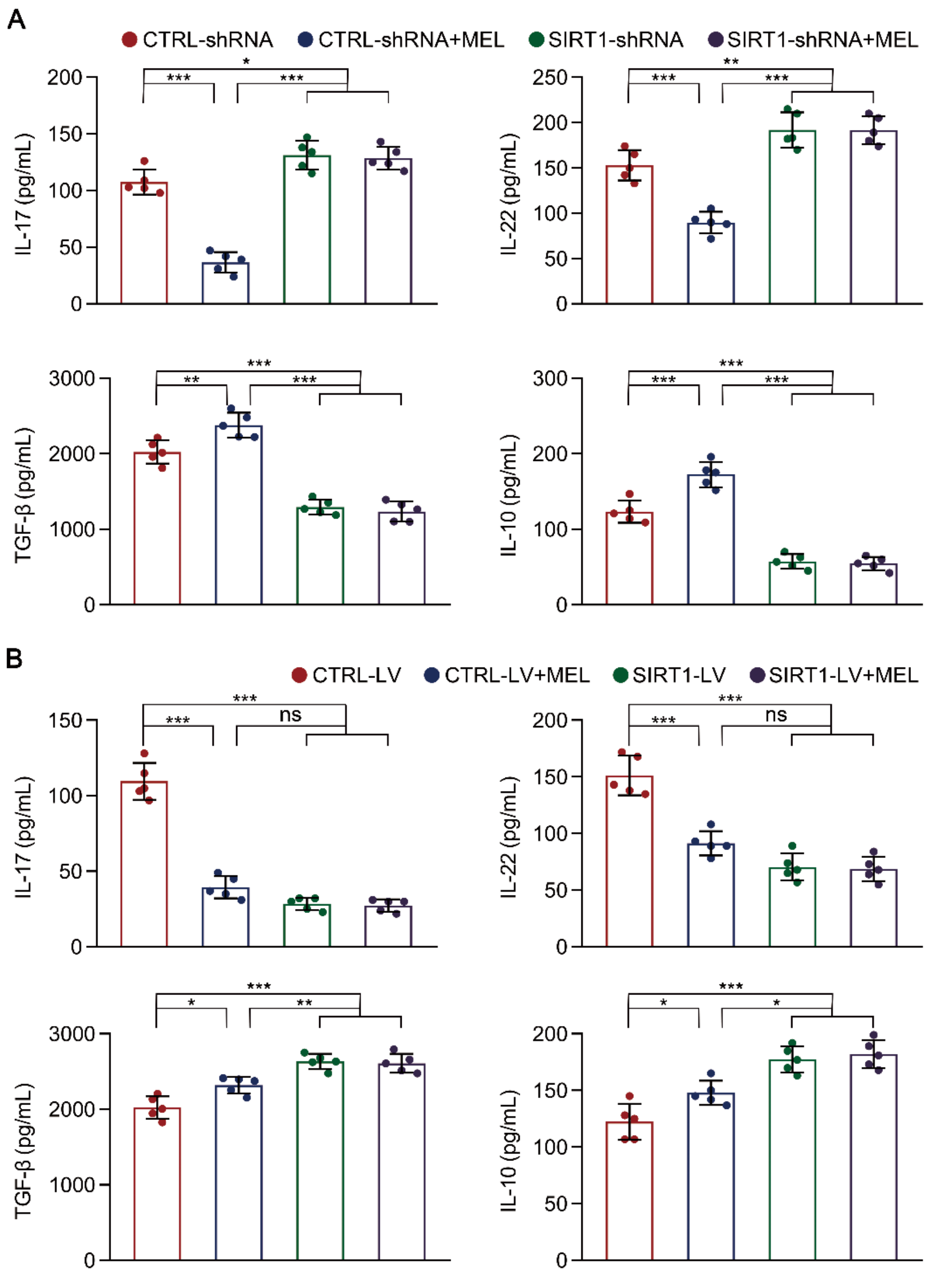

Supplementary Figure 6. Sorted naïve CD4$^+$ T cells from umbilical cord blood of healthy newborns were transduced with lentivirus-containing control-shRNA (CTRL shRNA) and SIRT1-shRNA (SIRT1 shRNA) (**A**), or control lentivirus (CTRL LV), and SIRT1-expressing lentivirus (SIRT1 LV) (**B**). At 24 h after transduction, CD4$^+$ T cells were differentiated under Th17 or Treg conditions for four days, in the presence of melatonin (20 ng/mL). Then the culture supernatants were collected and IL-17, IL-22, TGF-β, and IL-10 levels in the

supernatants were monitored by ELISA. Each symbol (**A, B**) represents an individual experiment (n = 5); column graphs represent the mean with error bars indicating standard deviation (SD), *P < 0.05; **P < 0.01; ***P < 0.001; ns: not significant. P values were derived through one-way ANOVA followed by the Bonferroni multiple comparison test (**A, B**).

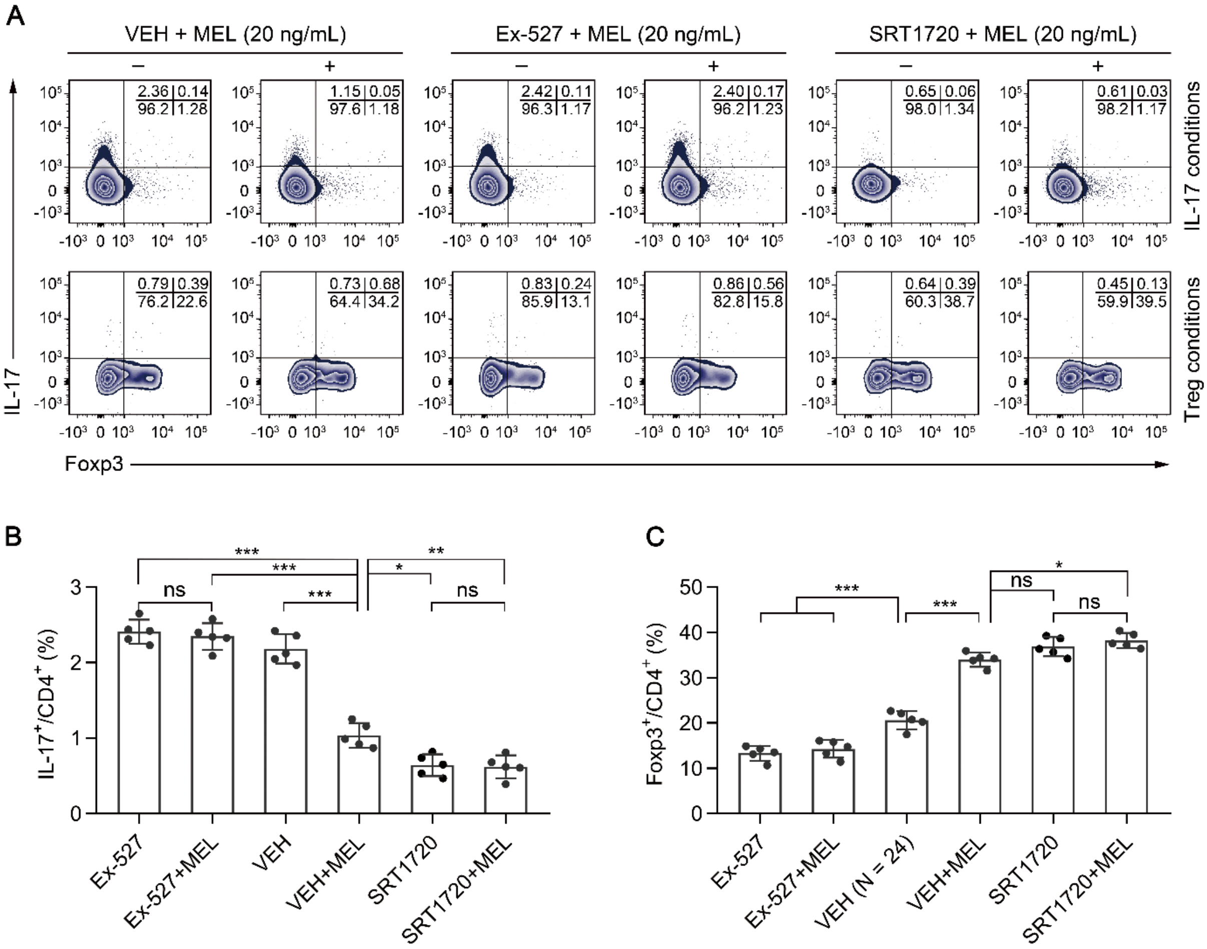

Supplementary Figure 7. Sorted naïve CD4$^+$ T cells of umbilical cord blood from healthy newborns were differentiated under Th17 or Treg conditions for 5 days, in the presence of vehicle (0.01% DMSO, VEH), melatonin (MEL), Ex-527, Ex-527+MEL, SRT1720, or SRT1720+MEL. Melatonin, Ex-527 (10 µM), SRT1720 (1 µM) and vehicle were added at the start of the cultures and at day 2. **(A)** Representative flow cytometry plots of IL-17 and Foxp3 expression in gated CD4$^+$ T cells under Th17 (top) and Treg (bottom) conditions. (**B, C)** Quantification of the frequency of Th17 (**B**) and Treg (**C**) cells in (**A**). Each symbol (**B**, **C**) represents an individual experiment (n = 5); column graphs represent the mean with error bars indicating standard deviation (SD), *$P < 0.05$; **$P < 0.01$; ***$P < 0.001$; ns: not significant.

P values were derived through one-way ANOVA followed by the Bonferroni multiple comparison test (**B**, **C**).

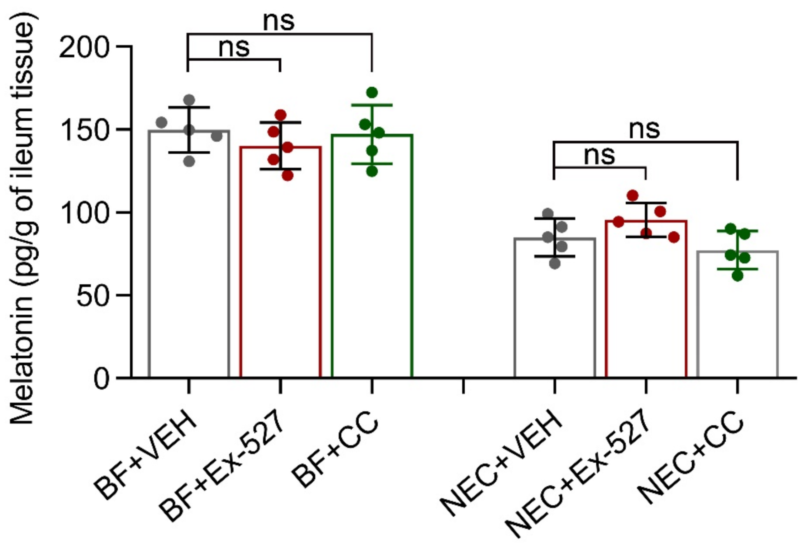

Supplementary Figure 8. ELISA analysis of the levels of melatonin in the small intestine of breastfed (BF) and necrotizing enterocolitis (NEC) pups with Ex-527, Compound C (CC) or vehicle (consisting of < 25% ethanol in PBS, VEH) treatment. Each symbol represents an individual experiment (n = 5); column graphs represent the mean with error bars indicating standard deviation (SD), ns: not significant. *P* values were derived through one-way ANOVA followed by the Bonferroni multiple comparison test.

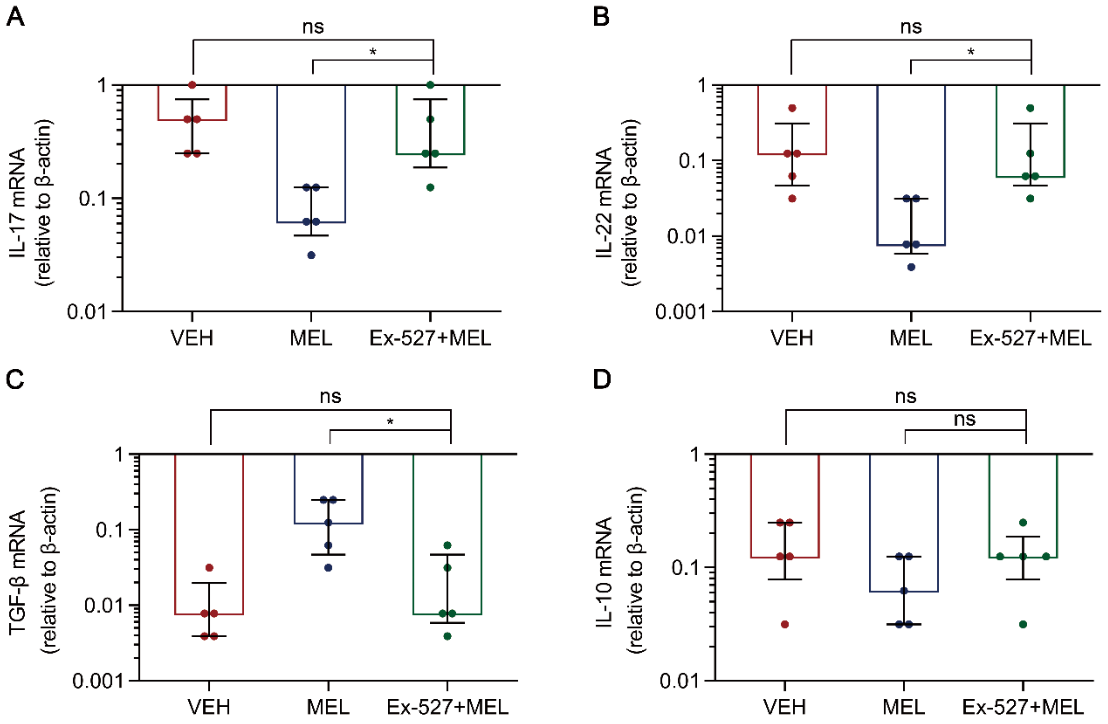

Supplementary Figure 9. (**A–D**) Real-time qRT-PCR analysis of relative mRNA expression of *IL-17* (**A**), *IL-22* (**B**), *TGF-β* (**C**) and *IL-10* (**D**) in ileum of NEC pups upon vehicle (consisting of < 25% ethanol in PBS, VEH) treatment, or treatment with melatonin (MEL) or melatonin combined with Ex-527 (MEL+Ex-527). Each symbol (**A–D**) represents an individual experiment (n = 5); column graphs represent the median with interquartile range, **P* < 0.05; ns: not significant. *P* values were derived through Kruskal-Wallis followed by the Pairwise Comparisons test (**A–D**).

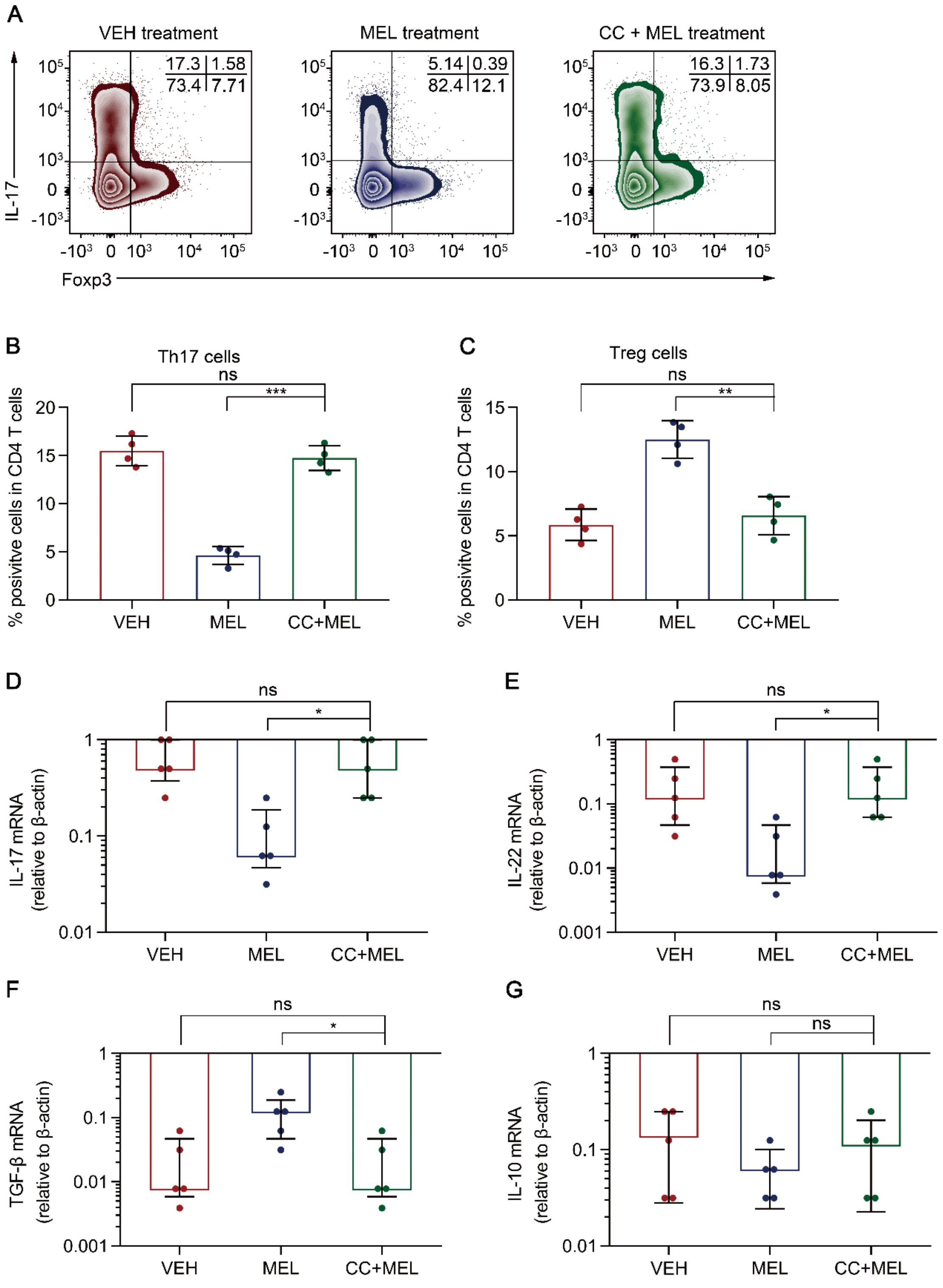

Supplementary Figure 10. AMPK inhibition attenuates maintenance of the Th17/Treg balance. **(A)** Representative flow cytometry plots of IL-17 and Foxp3 expression in gated

lamina propria $CD4^+$ T cells from ileum sections of pups following NEC induction upon treatment with vehicle (VEH) alone or with melatonin (MEL) or melatonin combined with Compound C (MEL + CC). **(B, C)** Quantification of the percentages of Th17 (**B**) and Treg (**C**) cells in **(A)**, n = 4 per group. (**D–G)** Real-time qRT-PCR analysis of relative mRNA expression of *IL-17* (**D**), *IL-22* (**E**), *TGF-β* (**F**) and *IL-10* (**G**) in ileum of VEH (n = 5), MEL (n = 5), and MEL + CC (n = 5) groups. Each symbol (**B–G**) represents an individual experiment; column graphs represent the mean with error bars indicating standard deviation (SD) (**B**, **C**), or median with interquartile range (**D–G**), $*P < 0.05$; $**P < 0.01$; $***P < 0.001$; ns: not significant. *P* values were derived through one-way ANOVA followed by Bonferroni multiple comparison test (**B**, **C**) or Kruskal-Wallis followed by the Pairwise Comparisons test (**D–G**).

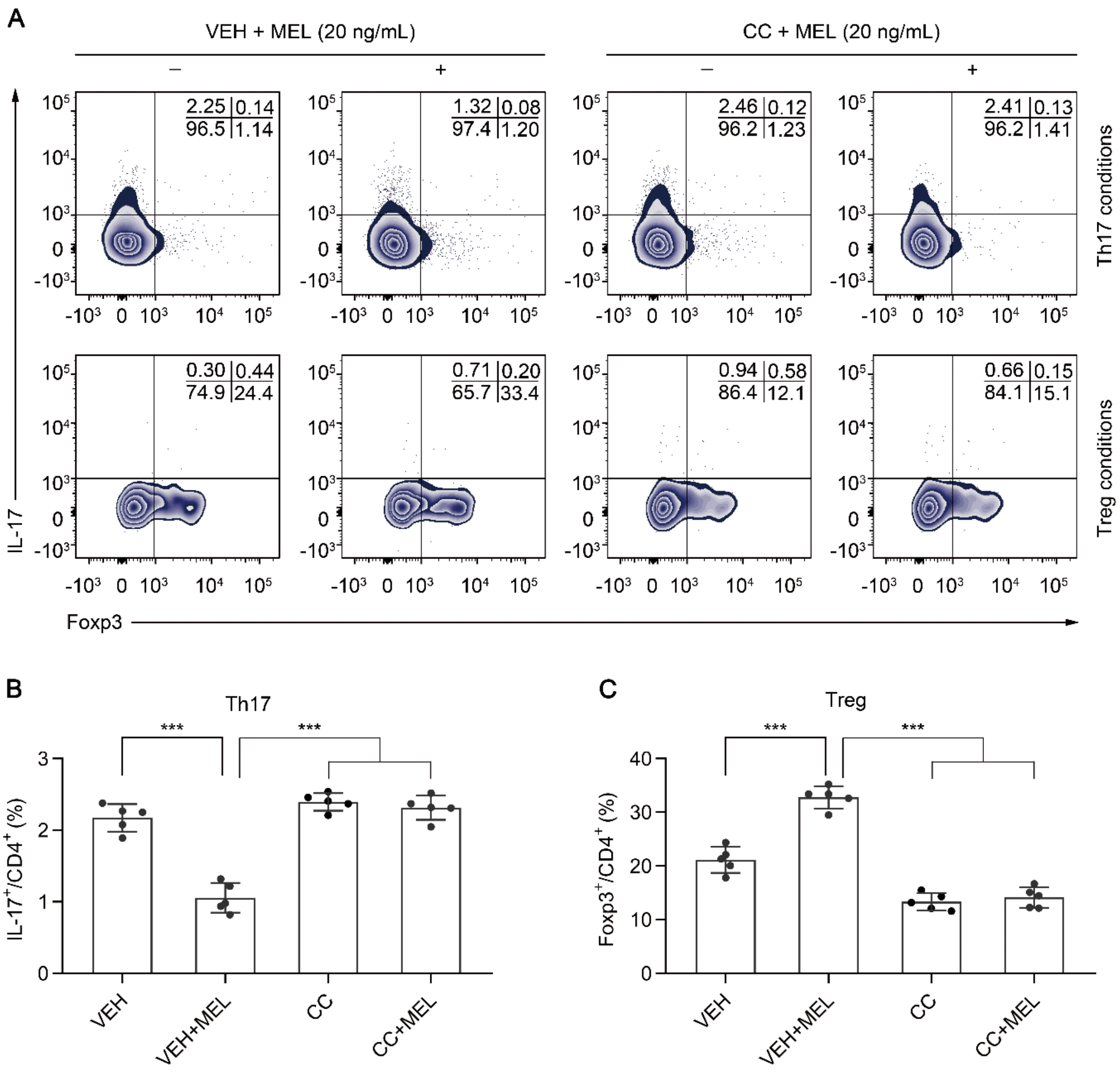

Supplementary Figure 11. Sorted naïve CD4$^+$ T cells of umbilical cord blood from healthy newborns were differentiated under Th17 or Treg conditions for 5 days, in the presence of vehicle (consisting of < 25% ethanol in PBS, VEH), melatonin (MEL), Compound C (CC), or MEL + CC. Melatonin, Compound C (1 μM) and vehicle were added at the start of the cultures and at day 2. **(A)** Representative flow cytometry plots of IL-17 and Foxp3 expression in gated CD4$^+$ T cells under Th17 (top) and Treg (bottom) conditions. **(B, C)** Quantification of the frequency of Th17 (**B**) and Treg (**C**) cells in (**A**). Each symbol (**B**, **C**) represents an

individual experiment (n = 5); column graphs represent the mean with error bars indicating standard deviation (SD), $^{***}P < 0.001$; ns: not significant. P values were derived through one-way ANOVA followed by the Bonferroni multiple comparison test (**B**, **C**).

Supplementary Table 1 Primers used for qRT-PCR in this study.

**Target gene**	**Primers**
**Human**	
Foxp3	Forward: 5’-CGTGACAGTTTCCCACAAGC-3’
	Reverse: 5’-GGTGGCATGGGGTTCAAG-3’
RORγt	Forward: 5’-GCTGGTTAGGATGTGCCG-3’
	Reverse: 5’-GAGTGGGAGAAGTCAAAGATGGA-3’
IL-10	Forward: 5’-ACCAAGACCCAGACATCAA-3’
	Reverse: 5’-CATTCTTCACCTGCTCCAC-3’
IL-17A	Forward: 5’-CTCGATTTCACATGCCTTCA-3’
	Reverse: 5’- GAGGGGCCTTAATCTCCAAA -3’
IL-22	Forward: 5’-GAGGAATGTGCAAAAGCTGA-3’
	Reverse: 5’-GCTTTGGGGCATCTAATTGT-3’
TGF-β	Forward: 5’-CACGATCATGTTGGACAACTGCTGC-3’
	Reverse: 5’-CTTCAGCTCCACAGAGAAGAACTGC-3’
SIRT1	Forward: 5’-GCAGATTAGTAGGCGGCTTG-3’
	Reverse: 5’-TCTGGCATGTCCCACTATCA-3’
β-actin	Forward: 5’-CCAGAGCAAGAGAGGCATCC-3’
	Reverse: 5’-TAGCACAGCCTGGATAGCAAC-3’
**Mouse**	CAGCAGACTCAATACACACCT
IL-10	Forward: 5’-TGGCCCAGAAATCAAGGAGG-3’
	Reverse: 5’-CAGCAGACTCAATACACACCT-3’
IL-17a	Forward: 5’- TTTAACTCCCTTGGCGCAAAA-3’
	Reverse: 5’- CTTTCCCTCCGCATTGACAC-3’
IL-22	Forward: 5’-CCGAGGAGTCAGTGCTAAGG-3’
	Reverse: 5’-TCTGGATGTTCTGGTCGTCA-3’
TGF-β	Forward: 5’-TGACGTCACTGGAGTTGTACGG-3’

	Reverse: 5’-GGTTCATGTCATGGATGGTGC-3’
β-actin	Forward: 5’-AACTCCATCATGAAGTGTGA-3’<br>Reverse: 5’-ACTCCTGCTTGCTGATCCAC-3’

127 **Supplementary Table 2 Antibodies for flow cytometry.**

**Reagents**	**Clone**	**Manufacturer**
**Human**		
Anti-CD3-FITC	UCHT1	BD Biosciences
Anti-CD3-APC	UCHT1	BD Biosciences
Anti-CD4- FITC	RPA-T4	BD Biosciences
Anti-CD4- APC/Cy7	RPA-T4	BD Biosciences
Anti-CD25- PE-Cy7	M-A251	BD Biosciences
Anti-CD45RA- PE	5H9	BD Biosciences
Anti- FoxP3-PE	259D/C7	BD Biosciences
Anti- IL-17A- PerCP-Cy5.5	N49-653	BD Biosciences
**Mouse**		
Anti-CD3- FITC	17A2	BD Biosciences
Anti-CD4- APC/Cy7	GK1.5	BioLegend
Anti-CD25- PE-Cy7	3C7	BioLegend
Anti-Foxp3- PE	MF23	BD Biosciences
Anti- IL-17A- PerCP-Cy5.5	TC11-18H10	BD Biosciences

128
